# Plasmonic Nanocavity-Induced Degradation Pathway of Boronic Acid Biosensing Interfaces Revealed by *In Situ* Tip-Enhanced Raman Spectroscopy

**DOI:** 10.1021/acsnano.6c05687

**Published:** 2026-06-23

**Authors:** Chengcheng Xu, Yuanzhi Xia, Julia Specht, Xiaokang Guan, Lyna Bourehil, Kim Greis, Robin N. Dürr, Victor Mougel, Naresh Kumar, Renato Zenobi

**Affiliations:** † Department of Chemistry and Applied Biosciences, 27219 ETH Zurich, Vladimir-Prelog-Weg 3, Zurich CH-8093, Switzerland; ‡ Department of Chemistry and the MOE Key Laboratory of Spectrochemical Analysis and Instrumentation, College of Chemistry and Chemical Engineering, 12466 Xiamen University, Xiamen, 361005 Fujian China

**Keywords:** boronic acid sensors, plasmonic nanocavity, plasmon-driven interfacial chemistry, nanoscale chemical transformation, surface degradation, tip-enhanced Raman spectroscopy (TERS)

## Abstract

Boronic acid-functionalized plasmonic interfaces enable ultrasensitive molecular recognition and biosensing in plasmonic nanocavities, yet their photochemical stability under hot-carrier excitation remains poorly understood. Here, we elucidate the plasmonic nanocavity-induced degradation pathway of self-assembled monolayers (SAMs) of 4-mercaptophenylboronic acid (4-MPBA) on Au(111) using hyperspectral tip-enhanced Raman spectroscopy (TERS). *In situ* TERS measurements visualize a stepwise plasmon-driven degradation process at the solid–air interface. Plasmonic excitation initiates deboronation and intermolecular cross-linking within the monolayer, followed by progressive oxidation at the sulfur center and eventual C–S bond cleavage. Complementary electrospray ionization mass spectrometry and X-ray photoelectron spectroscopy identify the final degradation products as oxidized sulfur species, consistent with the TERS spectral signatures. Temperature-programmed desorption mass spectrometry rules out thermal heating as the primary driving force, while density functional theory calculations support plasmon-mediated molecular activation via direct excitation or hot-electron transfer. Together, these results reveal a previously unrecognized degradation pathway of 4-MPBA SAMs on Au(111) that can be directly visualized within a plasmonic nanocavity, providing molecular-level insight into plasmon-driven interfacial chemistry and guiding the design of more stable boronic acid-based plasmonic biosensors.

## Graphical abstract

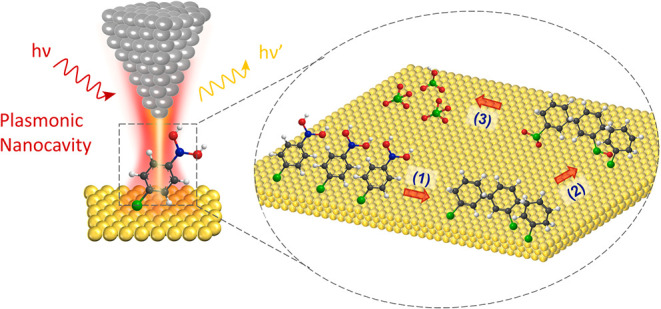

## Introduction

Covalent interactions between boronic acids and diols underpin a broad range of biomedical sensing strategies.^1,2^ In particular, boronic acid-based molecular probes have been widely employed for the selective recognition of diol-containing biomolecules, including saccharides^3,4^ and glycoconjugates,^5^ as well as carbohydrate-rich biological interfaces.^6^ In this context, 4-mercaptophenylboronic acid (4-MPBA) has emerged as a particularly versatile molecular probe, as its thiol group enables robust anchoring to plasmonic noble-metal surfaces, while the boronic acid functionality provides selective recognition of diol-containing biomolecules.^7^ This dual functionality has established 4-MPBA as a key component of boronic acid-functionalized plasmonic sensing platforms, where it operates simultaneously as a surface-immobilized molecular receptor and a spectroscopic reporter. Consequently, 4-MPBA self-assembled monolayers (SAMs) have become prototypical platforms for boronic acid-functionalized plasmonic biosensors. Accordingly, 4-MPBA has been implemented in plasmonic biosensors based on both ordered SAMs on Au surfaces and disordered SAM-like ligand shells on plasmonic Au and Ag nanoparticles, enabling the surface-enhanced Raman spectroscopy (SERS) detection of biologically relevant saccharides,^8^ complex lipid assemblies,^9^ and bacterial targets.^10^

When metallic nanostructures are separated by nanometer-scale gaps, plasmonic nanocavities form that further concentrate these fields into nanoscale hotspots capable of dramatically amplifying molecular signals and driving localized photochemical processes.^11,12^ However, the intense local electromagnetic fields that enable selective photochemical transformations may simultaneously drive unintended molecular degradation, which is an often-overlooked process that can compromise sensor performance and long-term stability.^13–15^ In particular, localized surface plasmon resonance (LSPR) excitation generates energetic hot carriers capable of inducing bond cleavage,^16,17^ molecular decomposition,^18,19^ and the formation of undesired byproducts.^20^ Gaining molecular-level insight into such degradation processes is therefore essential for the rational design of robust and reliable plasmonic sensors.^21,22^ Previous SERS studies on Au and Ag nanoparticle platforms have shown that 4-MPBA can undergo deboronation under plasmonic excitation, yielding thiophenol and/or biphenyl-4,4′-dithiol.^23,24^ However, the degradation pathway of 4-MPBA SAMs on well-defined Au(111) surfaces under plasmonic excitation has remained unexplored, largely due to the difficulty of detecting transient interfacial intermediates, which demand ultrahigh molecular sensitivity and specificity.

To probe such plasmon-driven interfacial processes, SERS^25–27^ and shell-isolated nanoparticle-enhanced Raman spectroscopy (SHINERS)^28,29^ are powerful methods of interfacial chemical analysis. SHINERS is particularly effective as it can provide strong Raman enhancement while minimizing direct chemical perturbation by the metal core.^30,31^ However, conventional SERS and SHINERS measurements typically report ensemble-averaged responses from many plasmonic hotspots or nanoparticle–surface junctions. As a result, they provide limited access to local heterogeneity, site-specific reaction pathways, and transient nanoscale intermediates. In contrast, tip-enhanced Raman spectroscopy (TERS) combines local plasmonic field enhancement with scanning probe microscopy, allowing chemical information to be acquired from a spatially defined tip–substrate nanocavity with nanometer-scale resolution.^32–39^ This capability makes TERS particularly well suited for resolving heterogeneous degradation processes at plasmonically active interfaces, where the local molecular environment, hotspot geometry, and reaction history can strongly influence the observed chemistry. TERS has been successfully employed to probe molecular decomposition,^40,41^ catalytic conversion,^42–47^ and molecular ordering^48–50^ within SAMs, providing unique insights into surface chemistry at the nanoscale. Its ability to directly track molecular structural changes through vibrational fingerprints with ultrahigh sensitivity makes TERS uniquely suited for investigating the degradation pathway of surface-bound boronic acid monolayers on Au(111).

Here, we employ *in situ* TERS to investigate the degradation pathway of 4-MPBA SAMs on Au(111) within a tip-induced plasmonic nanocavity. Hyperspectral TERS imaging with ∼ 5 nm resolution reveals a stepwise, plasmon-driven degradation process of the monolayer at the solid–air interface. Complementary analytical techniques, including electrospray ionization mass spectrometry (ESI-MS) and X-ray photoelectron spectroscopy (XPS), are employed to independently identify the chemical nature of the final degradation products, while temperature-programmed desorption mass spectrometry (TPD-MS) and density functional theory (DFT) calculations are used to disentangle plasmonic effects from purely thermal processes and to assess the feasibility of plasmon-mediated molecular activation. This correlative approach reveals a previously unrecognized degradation pathway in which surface-bound 4-MPBA undergoes intermolecular cross-linking and progressive sulfur oxidation, ultimately yielding predominantly inorganic sulfur oxide species on Au(111). These findings provide molecular-level insight into plasmon-driven interfacial chemistry and shed light on the stability limits of boronic acid-functionalized plasmonic biosensing interfaces.

## Results and Discussion

### TERS Nanocavity Platform for Monitoring On-Surface Degradation

To investigate the plasmon-driven degradation of 4-MPBA at the nanoscale, we employed a top-illumination STM-based TERS configuration capable of generating a highly confined plasmonic nanocavity between the metallic tip and the substrate. Figure 1 schematically depicts the scanning tunneling microscopy (STM)-based TERS setup used to investigate the on-surface degradation of 4-MPBA SAMs on Au(111), hereafter denoted as 4-MPBA/Au(111). STM imaging confirmed the atomically flat morphology of the Au(111) substrate (Figure S1), providing a well-defined surface for molecular adsorption. While no Raman signal was detected in the far field, TERS measurements revealed clear vibrational fingerprints of 4-MPBA, demonstrating monolayer-level sensitivity (Figure S2).

**Figure 1:**
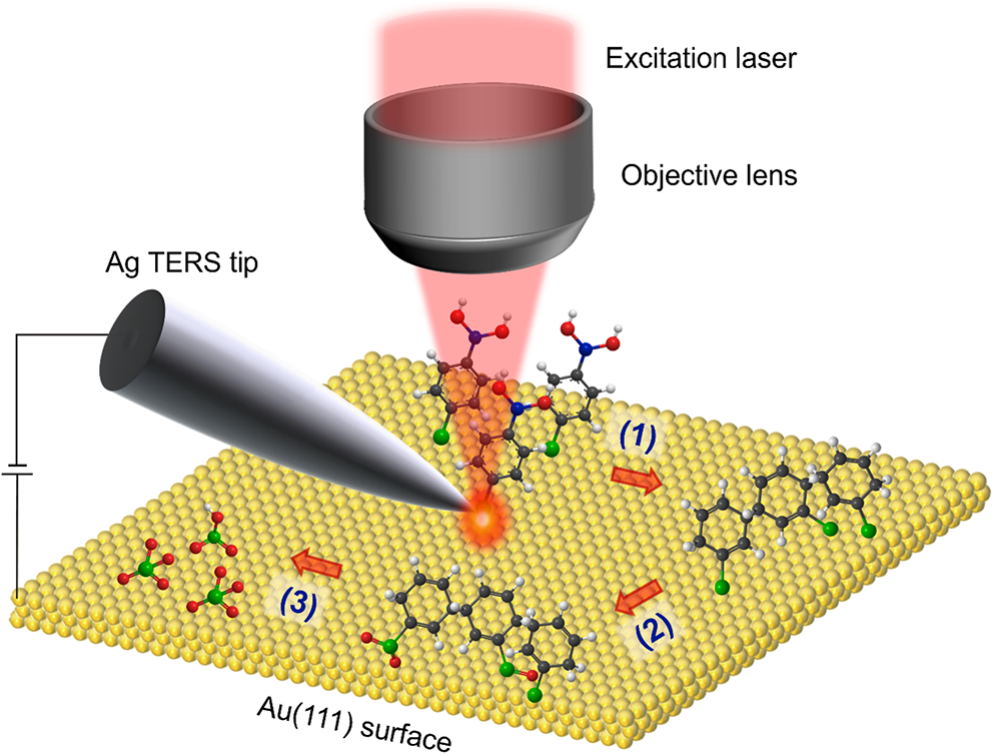
Schematic illustration of the top-illumination STM-TERS setup used to investigate the on-surface degradation of 4-MPBA on Au(111). Three stages of the 4-MPBA degradation pathway revealed by TERS measurements are depicted. Atom color scheme: yellow, gold; green, sulfur; gray, carbon; white, hydrogen; red, oxygen; and blue, boron.

### Hyperspectral TERS Imaging Reveals Stepwise Plasmon-Driven Degradation

Hyperspectral TERS mapping of 4-MPBA/Au(111) performed at a low laser power of 65 μW yielded highly reproducible spectra across the mapped area. A representative waterfall plot of 100 spectra and the corresponding averaged spectrum are shown in Figure 2a,2b, respectively. Both display two dominant vibrational modes at 1587 and 1075 cm^–1^, assigned to the phenyl ring C=C stretching mode (ν_C=C_) and the coupled β_C–C–C_ + ν_C–S_ vibration, consistent with pristine 4-MPBA SAMs.^51^ Upon increasing the laser power to 2.5 mW (38.5-fold increase) and probing the same surface region, the characteristic molecular bands of 4-MPBA disappear (Figure 2c,d). Instead, a pronounced band emerges at 978 cm^–1^, attributed to the S=O stretching vibration (ν_S=O_).^52^ The appearance of this mode indicates laser-induced chemical transformation of the adsorbed 4-MPBA into sulfur oxide species, confirming its on-surface degradation under plasmonic excitation.

**Figure 2:**
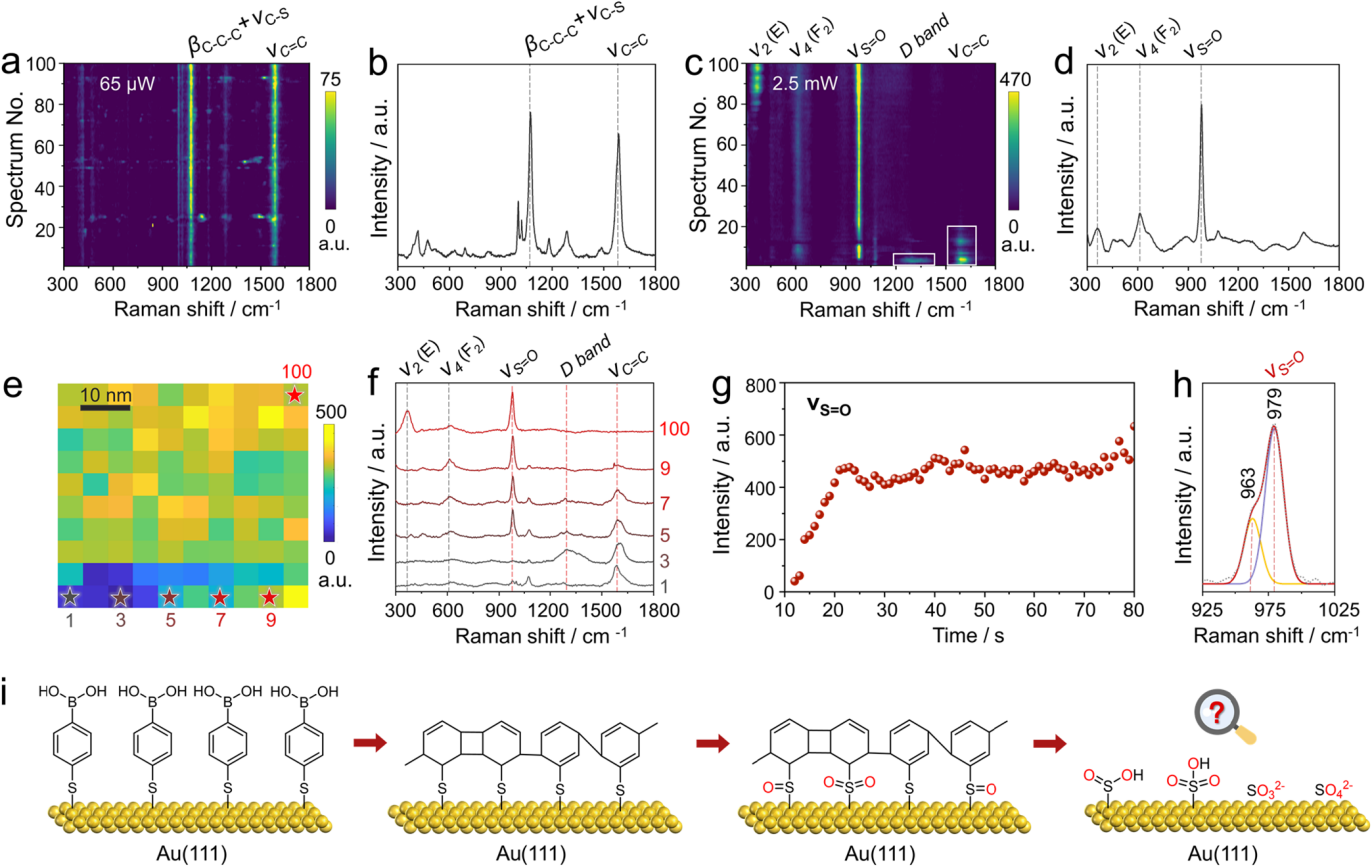
Monitoring of plasmon-induced on-surface degradation of 4-MPBA/Au(111). (a) Waterfall plot comprising 100 individual TERS spectra acquired from a hyperspectral TERS map at a laser power of 65 μW. (b) Corresponding averaged TERS spectrum derived from the data shown in panel (a). (c) Waterfall plot comprising 100 individual TERS spectra acquired from a hyperspectral map at an increased laser power of 2.5 mW measured in the same area as panel (a). (d) Corresponding averaged TERS spectrum derived from the data shown in panel (c). For the TERS maps in panels (a) and (c), the integration time was 1 s per spectrum, the mapped area was 50 × 50 nm^2^, and the step size was 5 nm. (e) Hyperspectral TERS intensity map of the 978 cm^–1^ vibrational band derived from the data shown in panel (c). (f) Representative TERS spectra extracted from six distinct locations within the map shown in panel (e), corresponding to different stages of 4-MPBA degradation. (g) Time-dependent evolution of the S=O stretching band intensity derived from the hyperspectral TERS data in panel (e). (h) Gaussian deconvolution of the S=O stretching region, revealing two sub-bands at 963 and 979 cm^–1^. (i) Schematic representations of molecular configurations along the identified degradation pathway (left to right): pristine 4-MPBA; deboronation and intermolecular phenyl ring cross-linking; oxidation of sulfur; and formation of highly oxidized products after C–S bond cleavage.

Figure 2e shows a hyperspectral TERS intensity map of the 978 cm^–1^ band, with six representative TERS spectra extracted from distinct locations within the map presented in Figure 2f. The time-dependent evolution of the S=O stretching band intensity derived from the hyperspectral data set is plotted in Figure 2g. Collectively, these data provide direct spectroscopic evidence for the progressive laser-induced degradation of 4-MPBA/Au(111). As illustrated in Figure 2f, increasing laser irradiation leads to a gradual attenuation and eventual disappearance of the characteristic 4-MPBA vibrational modes at 1075 and 1587 cm^–1^, accompanied by a concomitant increase in the intensity of the ν_S=O_ mode, which is quantitatively captured in the time-resolved intensity profile shown in Figure 2g. The new bands at 380 and 610 cm^–1^ are assigned to the low-frequency S–O deformation modes of oxidized sulfur species. This assignment is consistent with reported Raman signatures of sulfate-containing compounds, in which the ν_2_(E) deformation modes appear in the 360–450 cm^–1^ region and the ν_4_(F_2_) bending modes occur in the 610–650 cm^–1^ region.^52–54^

The spectra in Figure 2 were background-subtracted to facilitate visualization and vibrational assignment. To verify that the observed evolution of the ν_S=O_ band is not introduced by this procedure, we analyzed the corresponding raw, not background-subtracted hyperspectral TERS spectra (Figure S3). The raw spectra show the same overall increase in ν_S=O_ intensity, confirming that the emergence of the oxidized sulfur signal is not an artifact of background subtraction. We further extracted the broad continuum background from representative raw spectra acquired at different irradiation times and compared the spectral position of its maximum with the ν_S=O_ intensity evolution (Figure S3b–d). During irradiation, the background maximum blue-shifts toward shorter wavelengths and evolves in parallel with the increasing ν_S=O_ intensity. This correlation suggests that the local plasmonic response of the tip–substrate nanocavity changes during degradation, potentially enhancing local-field strength and hot-carrier generation and thereby promoting oxidation at the sulfur center.

Importantly, both the waterfall plot in Figure 2c and the representative spectra in Figure 2f reveal an unexpected intermediate spectral regime at the early stages of laser irradiation. In this regime, the spectra are dominated by broad bands centered at approximately 1350 and 1620 cm^–1^, while the characteristic 4-MPBA modes and the S=O stretching band are absent. These broad features closely resemble the D, D′, and G bands typically observed in highly disordered graphene.^55,56^ Their appearance suggests the formation of a transient intermediate state in which neighboring phenyl rings within the SAM undergo extensive cross-linking, giving rise to a highly disordered sp^2^-hybridized carbonaceous network.^20^ The emergence of the D-band-like feature indicates a high density of structural defects, analogous to disordered carbon networks formed in single-layer graphene under heavy ion irradiation.^57,58^ Note that due to the 1 s acquisition time of the spectra in the time-dependent TERS measurements, transient intermediates with lifetimes shorter than 1 s or species undergoing rapid desorption from the Au surface may not be captured. Future implementation of high-speed TERS approaches with improved temporal resolution may enable access to such subsecond or short-lived intermediates.

Control TERS experiments under an inert N_2_ atmosphere further clarify the role of the surrounding environment in the degradation pathway, as shown in Figure S4. Under ambient conditions, high-power irradiation produces a pronounced ν_S=O_ band, indicating formation of oxidized sulfur species. In contrast, under N_2_, distinct ν_S=O_ signatures are strongly suppressed, whereas D-/G-band-like features remain observable. These results indicate that atmospheric oxygen is required for efficient sulfur oxidation, while plasmon-induced deboronation and intermolecular cross-linking can still proceed under oxygen-limited conditions.

Notably, the extent of molecular cross-linking under plasmonic excitation depends sensitively on the incident laser power, as evidenced by the laser-power-dependent TERS measurements of 4-MPBA/Au(111) shown in Figure S5. Cross-linking is not observed at low excitation powers and initiates only when the laser power exceeds 0.30 mW, becoming pronounced at powers above 0.98 mW. Previous SERS studies of 4-MPBA adsorbed on Au and Ag nanoparticles have shown that, under high laser-power irradiation, 4-MPBA undergoes deboronation, yielding benzenethiol or biphenyl-4,4′-dithiol species.^23,24^ In contrast, our measurements show that upon laser irradiation above 1.1 mW, the characteristic 4-MPBA vibrational modes at 1075 and 1587 cm^–1^ disappear, accompanied by the emergence of broad D- and G-band-like features (Figures 2 and S5). These observations indicate that the deboronation of 4-MPBA is followed by extensive cross-linking between adjacent phenyl rings within the SAM on Au(111).

The degradation pathway of 4-MPBA/Au(111) under laser irradiation was reproducibly observed on an independent sample using a different TERS tip, as shown in Figures S6 and S7. While the pristine 4-MPBA adlayer remains intact under low-power irradiation (65 μW), it undergoes progressive degradation to oxidized sulfur species at higher laser power (2.5 mW). Consistent with the data presented in Figure 2, the transformation proceeds via an intermediate cross-linked structure formed following the deboronation of the 4-MPBA molecules. The molecular configurations along the degradation pathway of 4-MPBA on Au(111) revealed by the TERS measurements are schematically summarized in Figure 2i.

To evaluate the early-stage degradation behavior of 4-MPBA, we performed a ratiometric analysis of two independent hyperspectral TERS data sets, following the approach reported by Shen et al.^59^ The procedure and fitting results are provided in Supporting Note 1 and Figure S8. This analysis yielded apparent early-stage degradation rate constants of 0.91 and 0.61 s^–1^ for the data sets shown in Figures 2e and S7a, respectively. Even though these values are semiquantitative apparent rates rather than absolute kinetic constants, they provide a useful measure of the rapid initial loss of pristine 4-MPBA relative to the formation of oxidized sulfur species under high-power plasmonic excitation.

To test whether plasmon-induced sulfur oxidation is a general response of thiolated SAMs or a molecule-specific degradation channel of 4-MPBA, we performed additional control TERS measurements on thiophenol and 4-biphenylthiol (4-BPT) SAMs on Au(111) under the same hyperspectral imaging conditions used for the 4-MPBA experiments (Figures S9 and S10 and Supporting Information, Note 2). Thiophenol exhibited weak signatures of sulfur oxidation at high laser power, whereas 4-BPT largely retained its molecular integrity. These results indicate that sulfur oxidation is not unique to 4-MPBA but that its efficiency might be influenced by the molecular structure, packing density, and intermolecular ordering.^60,61^

The emergence of a single dominant band at 978 cm^–1^ raises the question of the precise chemical nature of the oxidized sulfur species formed following the degradation of 4-MPBA on Au(111). Figure 2h presents an expanded view of the spectral region surrounding this feature. Gaussian deconvolution reveals that the apparent single band comprises two overlapping components: a dominant peak centered at 979 cm^–1^ and a lower-frequency shoulder at 963 cm^–1^. The Raman frequency of the S=O stretching vibration is known to be sensitive to the oxidation state of sulfur.^62^ For example, in solution-phase measurements, sulfite species (SO_3_^2–^) typically exhibit symmetric S=O stretching modes in the range of 960–970 cm^–1^,^63^ whereas sulfate species (SO_4_^2–^) appear at higher wavenumbers, typically between 980 and 992 cm^–1^.^54,64^ Accordingly, the observed band splitting indicates the coexistence of oxidized sulfur species with at least two distinct oxidation states. Four plausible sulfur-containing structures corresponding to this final stage of degradation are illustrated in Figure 2i. However, additional experimental analysis is required to unambiguously assign the individual species contributing to the observed S=O stretching bands.

### Chemical Identification of the Degradation Products at the Solid–Air Interface

To achieve a more definitive chemical identification of the oxidized sulfur species, we performed complementary TERS, XPS, and ESI-MS measurements. Because 4-MPBA degradation occurs exclusively within the highly localized TERS near-field, which is confined to the nanoscale, it is not feasible to collect sufficient quantities of degraded material from the Au(111) surface for conventional XPS or ESI-MS analysis. To circumvent this limitation, we adopted a strategy in which the entire 4-MPBA SAM was oxidized using UV-ozone treatment, an approach previously reported for the oxidation of decanethiol SAMs.^65^ A waterfall plot of TERS spectra acquired from a hyperspectral map of 4-MPBA/Au(111) after 30 min of UV-ozone exposure is shown in Figure 3a, which exhibits a dominant band at 978 cm^–1^ accompanied by a shoulder at 963 cm^–1^ (Figure 3b,c). These spectral features closely match those observed for the degradation products formed during high-power TERS measurements (Figure 2d,h), indicating that the UV-ozone treatment produces similar oxidized sulfur species as those generated under intense plasmonic excitation.

**Figure 3:**
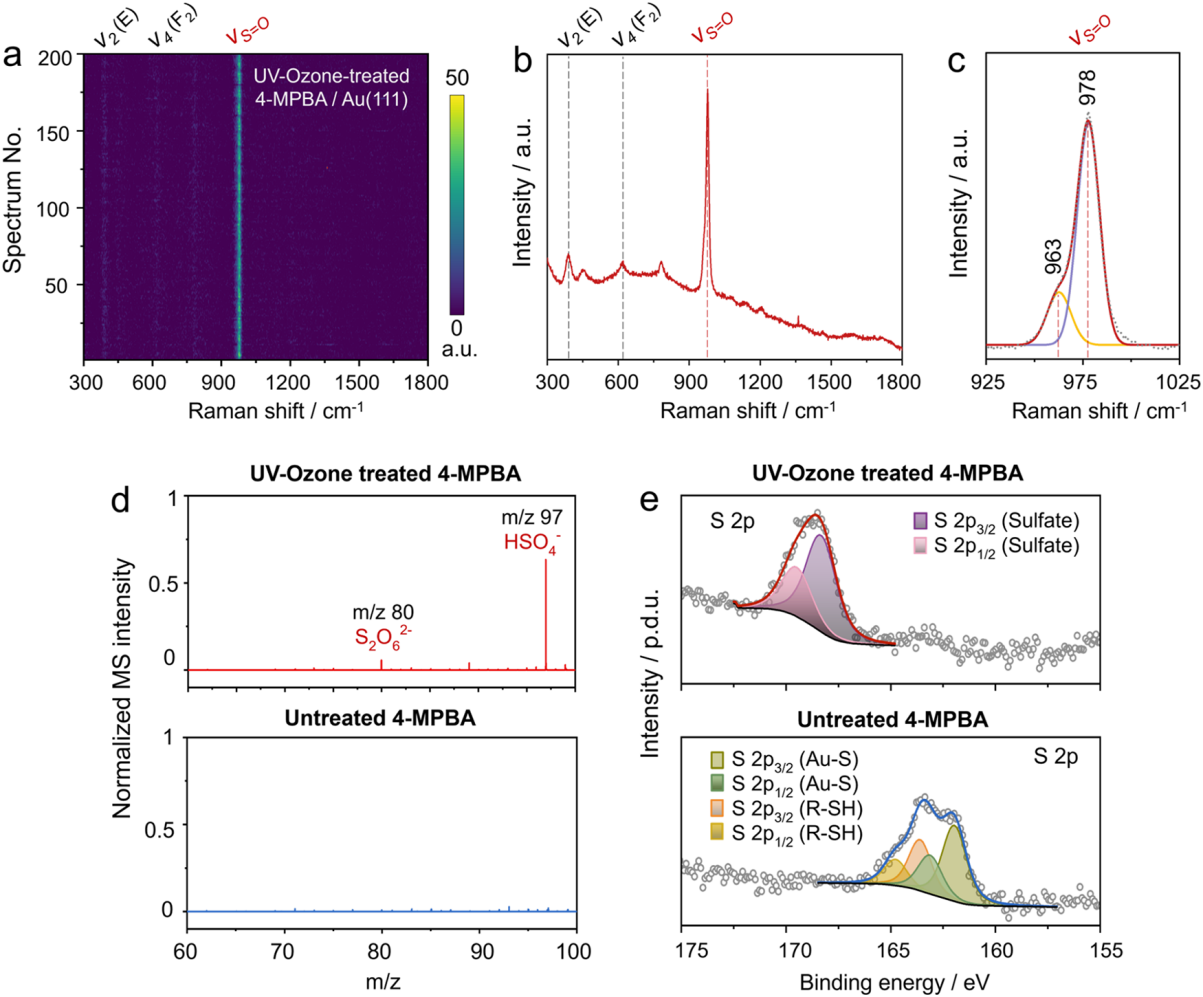
(a) Waterfall plot comprising 200 individual spectra acquired from a hyperspectral TERS map of ozone-treated 4-MPBA/Au(111). (b) Corresponding averaged TERS spectrum derived from the data shown in panel (a). (c) Gaussian peak fitting of the S = O stretching band, showing two peaks at 963 and 978 cm^–1^. (d) Negative-mode ESI mass spectra: top, UV-ozone-treated 4-MPBA recovered from Au(111) surface using ammonium acetate buffer; bottom, untreated 4-MPBA used as a reference. (e) High-resolution S 2p XPS spectra: top, UV-ozone-treated 4-MPBA/Au(111) surface; bottom, untreated 4-MPBA/Au(111).

The oxidized thiol products were collected by rinsing the UV-ozone-treated 4-MPBA SAM with 10 μL of ammonium acetate buffer and subsequently analyzed by ESI-MS.^66,67^ The resulting mass spectrum (Figure 3d, **top panel**) exhibits a strong ion signal at *m*/*z* = 97 and a weaker signal at *m*/*z* = 80, which are assigned to HSO_4_^–^ and S_2_O_6_^2–^, respectively. The presence of HSO_4_^–^ indicates complete oxidation of surface-bound sulfur to sulfate (SO_4_^2–^) on Au(111), whereas the S_2_O_6_^2–^ signal likely arises from a coupling reaction of partially oxidized sulfur intermediates (e.g., Au–SO_3_H/SO_3_^2–^) during dissolution in ammonium acetate buffer. By contrast, the mass spectrum of the unoxidized 4-MPBA sample (Figure 3d, **bottom panel**) shows none of these features, confirming that the observed HSO_4_^–^ and S_2_O_6_^2–^ ions originate from the oxidative transformation of the 4-MPBA SAM.

High-resolution S 2p XPS spectra of 4-MPBA/Au(111) before and after UV-ozone treatment are shown in Figure 3e (bottom and top panels, respectively). The untreated sample exhibits two S 2p doublets: one at 162.0 eV (S 2p_3/2_) and 163.1 eV (S 2p_1/2_), consistent with thiolate-bound sulfur,^68,69^ and a second at 163.6 eV (S 2p_3/2_) and 164.8 eV (S 2p_1/2_), attributed to unbound^70^ or physisorbed dimerized thiols.^71^ Upon UV-ozone treatment, the S 2p signal shifts markedly to higher binding energies and is well described by a single doublet at 168.3 eV (S 2p_3/2_) and 169.5 eV (S 2p_1/2_). These values (Table S1) fall between those of the Na_2_SO_3_ and Na_2_SO_4_ reference compounds (Figure S11) but are consistent with reported sulfate species.^72^ These results indicate that the UV-ozone treatment predominantly converts surface-bound 4-MPBA molecules to sulfate species, while the presence of sulfite species cannot be unambiguously resolved by XPS. Indeed, partial oxidation of Na_2_SO_3_ to sulfate is evident in the reference spectra (Figure S11), suggesting that any sulfite species formed may undergo further oxidation, thereby complicating their detection. Complementary high-resolution B 1s and C 1s XPS measurements (Figure S12) show substantial loss of boron and a pronounced decrease in the carbon signal, respectively, consistent with deboronation and C–S bond cleavage observed during the laser-induced degradation of 4-MPBA.

### Evaluating Thermal versus Plasmon-Driven Degradation Pathways

The on-surface conversion of 4-MPBA into sulfite and sulfate species is, to our knowledge, unprecedented. This raises the key question of whether the laser-induced degradation is driven by thermal heating or by plasmonically generated hot carriers, as increased laser power can enhance both effects within the TERS near-field. In our previous study, we estimated that under 2.5 mW laser irradiation the temperature in the TERS near-field does not exceed 420 K,^20^ whereas it was found that 4-MPBA had completely desorbed from a Au surface after heating it for 10 min to 448 K.^23^ Therefore, to directly evaluate the contribution of thermal effects, we performed TPD-MS measurements on 4-MPBA/Au(111) to identify heat-induced reaction products in the absence of plasmonic excitation (Figure 4). The schematic illustration of the TPD-MS measurement setup is shown in Figure S13, while temperature-dependent traces for selected ions are presented in Figure S14. As shown in Figure 4e,f, thermal heating alone does not give rise to the oxidized sulfur species identified as degradation products in the TERS experiments (Figure 2). Instead, heating predominantly leads to the formation of dehydrated 4-MPBA (Figure 4b) and dimerized (Figure 4c,d) species, together with desorption of intact 4-MPBA (Figure 4a), whose spectral signatures are not observed in the TERS measurements. These results indicate that the degradation pathway of 4-MPBA resolved by TERS cannot be explained by thermal activation alone.

**Figure 4:**
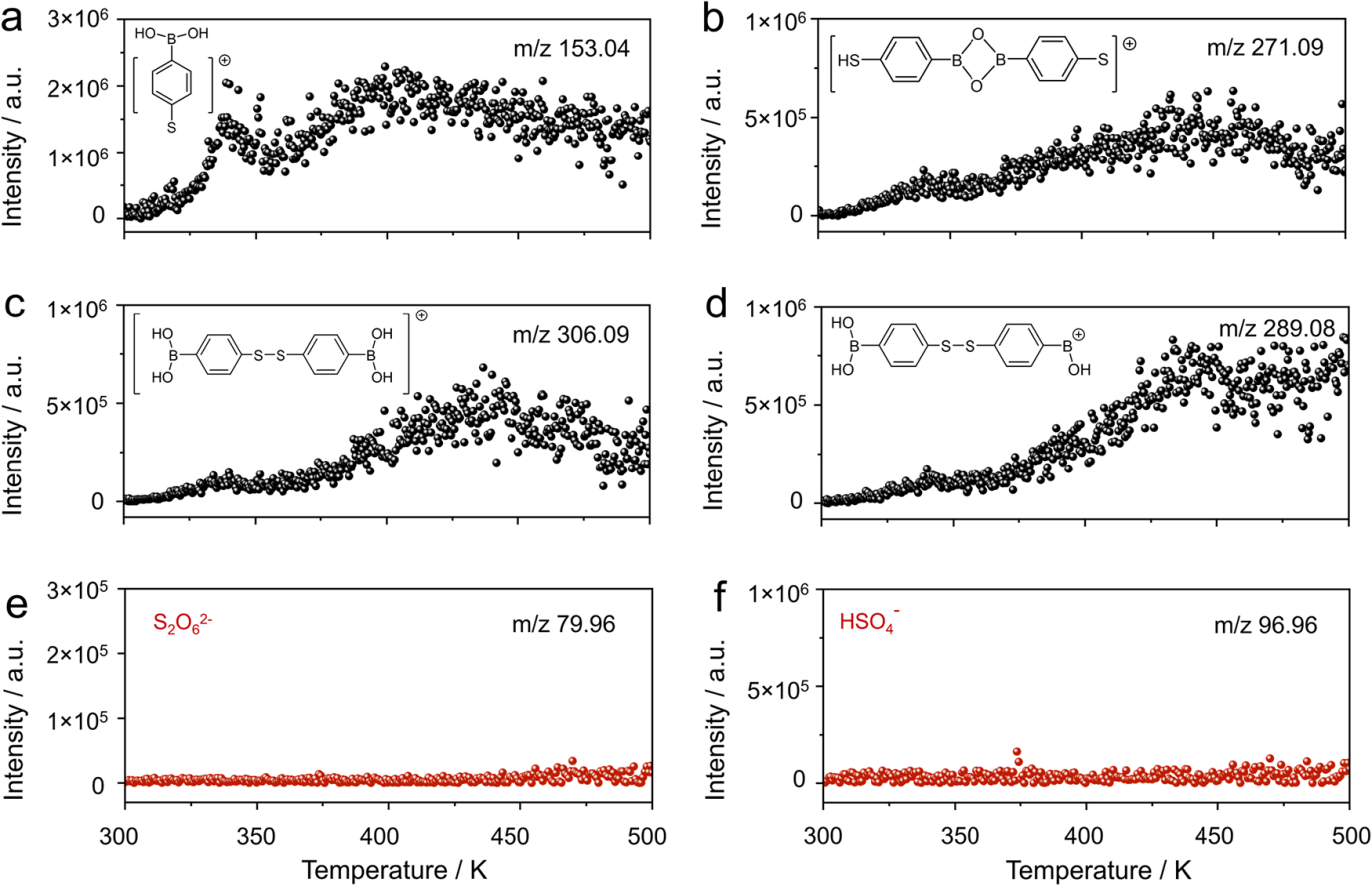
(a–f) TPD-MS traces of selected desorbed species recorded between 300 and 500 K, with the corresponding molecular structures shown as insets. The TPD-MS data indicate that thermal activation induces the following surface reactions: (a) cleavage of the Au–S bond in 4-MPBA/Au(111); (b) formation of dehydrated species via condensation reactions; and (c, d) generation of dimerized products through thiol–thiol coupling. Notably, no oxidized sulfur species were detected under purely thermal conditions (e, f).

### DFT Analysis of Plasmon-Mediated Molecular Activation

Plasmon-driven reactions can proceed either via hot-carrier transfer from the metal to the adsorbate or through direct plasmon-induced electronic excitation of the molecule.^74^ To evaluate the feasibility of these pathways in the plasmonic TERS near-field, we performed DFT calculations of the electronic structure of Au-bound 4-MPBA. The calculated frontier orbital energies, summarized in Figure 5, place the highest occupied molecular orbital (HOMO) at −5.69 eV and the lowest unoccupied molecular orbital (LUMO) at −3.88 eV, corresponding to a HOMO–LUMO gap of 1.81 eV. When referenced to the −5.35 eV^73^ Fermi level of Au(111) in vacuum, these energetic alignments indicate that localized surface plasmons excited by 633 nm laser irradiation (photon energy 1.96 eV) can efficiently activate adsorbed 4-MPBA molecules, via both indirect hot-electron transfer as well as direct plasmon-induced electronic excitation.

**Figure 5:**
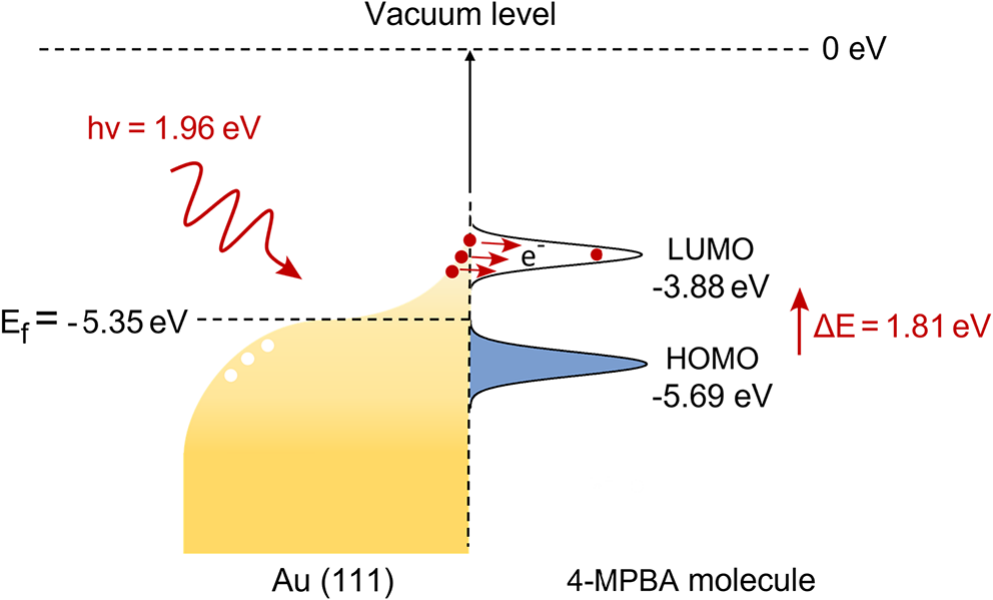
Plasmonic excitation scheme for a 4-MPBA molecule adsorbed on Au(111). To simplify the electronic structure analysis, a model system consisting of a single 4-MPBA molecule coordinated to three Au atoms in vacuum was employed to calculate the HOMO–LUMO energy gap. Within this model, the calculated HOMO and LUMO energies are −5.69 and −3.88 eV, respectively, corresponding to a HOMO–LUMO gap of 1.81 eV. The Fermi level of Au(111) in vacuum was taken as −5.35 eV.^73^ These energetic considerations indicate that localized surface plasmons (LSPs) generated under 633 nm laser excitation (photon energy 1.96 eV) can activate 4-MPBA on Au(111), either via indirect hot-electron transfer from the metal or through direct plasmon-induced electronic excitation.

Additional evidence that the degradation pathway of 4-MPBA is plasmon-driven is provided by the observation of cross-linked intermediate structures, manifested by the emergence of broad D- and G-band-like features upon high-power irradiation of the 4-MPBA adlayer. In our previous work, we used TERS to demonstrate that such cross-linking is driven by plasmon-generated hot carriers rather than by thermal heating.^20^ Consistent with these findings, the appearance of cross-linked intermediates here indicates that the degradation pathway of 4-MPBA identified in this study is likewise predominantly plasmon-mediated. In this mechanism, plasmonic excitation activates 4-MPBA molecules, promote deboronation and cross-linking between adjacent molecules within the adlayer, which ultimately leads to the formation of oxidized sulfur species.

It is important to emphasize that elucidation of this previously unreported degradation pathway is enabled uniquely by hyperspectral TERS measurements. The combination of an atomically flat Au(111) substrate, a well-defined 4-MPBA SAM, and a single, spatially confined, nanoscopic plasmonic hotspot at the TERS tip apex provides a level of experimental control not accessible in conventional SERS experiments, which typically rely on ensemble averaging over heterogeneous plasmonic nanostructures. These controlled conditions yield reproducible, site-specific spectra and allow an unambiguous, nanoscale-level view of the degradation processes of 4-MPBA. The present results provide practical guidelines for improving the stability of 4-MPBA-based plasmonic sensing interfaces. The laser-power-dependent TERS measurements identify an excitation regime in which plasmon-induced degradation becomes pronounced under the present nanocavity conditions, while the N_2_-control experiments show that oxygen is required for efficient sulfur oxidation. These findings suggest that degradation can be mitigated by minimizing the optical dose, reducing acquisition dwell times, and optimizing plasmonic substrates to achieve sufficient Raman enhancement at lower incident powers. In parallel, improving monolayer packing and reducing defects at the Au–S interface may limit oxygen access and suppress sulfur oxidation. More broadly, these results emphasize that the design of boronic acid-functionalized plasmonic probes must balance three coupled factors: high Raman enhancement, efficient molecular recognition, and resistance to hot-carrier- and oxygen-assisted degradation.

## Conclusions

In summary, we have investigated a plasmon-driven degradation pathway of 4-MPBA on Au(111) using a combination of hyperspectral TERS, ESI-MS, XPS, TPD-MS, and DFT calculations. Under 2.5 mW laser irradiation, 4-MPBA molecules undergo a sequence of transformations within the plasmonic TERS nanocavity, comprising deboronation, intermolecular cross-linking, oxidation at the sulfur center, and ultimately C–S bond cleavage, leading to the formation of oxidized sulfur species. The chemical identity of the final degradation products was established by ESI-MS and XPS, which identify SO_4_^2–^ as the dominant species with minor contributions from −SO_3_H/SO_3_^2–^, in agreement with the TERS-based spectral assignments. Control experiments using TPD-MS rule out thermal heating as the primary driving force for 4-MPBA degradation. Instead, DFT calculations support the feasibility of plasmonic activation of adsorbed 4-MPBA, either via direct electronic excitation or indirect hot-electron transfer, which initiates the observed degradation pathway. These findings reveal a previously unrecognized degradation mechanism of 4-MPBA on plasmonic metal surfaces and underscore the importance of chemical stability under plasmonic excitation in boronic acid-based molecular probes. More broadly, this work provides critical molecular-level insight into the photochemical robustness of 4-MPBA under plasmonic excitation, with direct implications for the design, optimization, and long-term reliability of 4-MPBA-based plasmonic biosensors.

## Methods and Experiments

### Sample Preparation

4-Mercaptophenylboronic acid (4-MPBA) was purchased from Sigma-Aldrich and used without further purification. A 10 mM 4-MPBA solution was prepared in ethanol. Au(111) on mica was used as the substrate for the preparation of 4-MPBA SAMs. The Au(111) surface was immersed in 10 mM 4-MPBA overnight and then thoroughly rinsed with ethanol. Freshly prepared samples were dried under ambient conditions and used for the TERS measurements.

### Oxidation of 4-MPBA Adlayers

#### UV-Ozone Treatment

The 4-MPBA/Au(111) samples were placed in a commercial UV-ozone device (BioForce Nanosciences, USA) for 30 min. The freshly treated samples were used directly for TERS and XPS measurements. For ESI-MS analysis, the surface products were extracted by rinsing the Au(111) surface with 10 μL of ammonium acetate buffer, and the resulting solution was analyzed by ESI-MS.

### TERS Tip Preparation

All TERS tips were fabricated using a home-built electrochemical etching setup. A platinum wire (0.25 mm in diameter) was shaped into a ring of approximately 13 mm in diameter to serve as the cathode. High-purity silver wire (99.99%, 0.25 mm in diameter) was used as the anode and positioned in the center of the platinum ring. The etching solution consisted of perchloric acid and ethanol mixed at a volume ratio of 4:1. A potential was applied between the electrodes using a custom circuit, and the etching terminated automatically after the etched Ag wire segment detached into the solution. The etched tips were subsequently rinsed in ethanol and checked under a Nikon stereo microscope at 360× magnification. The diameter of the TERS tip apex is estimated to be 80 nm.^75^

### TERS Measurements

The system consists of a hybrid STM scanning head for top-illumination (NT-MDT, Russia) integrated with a Raman spectrometer (NTEGRA Spectra Upright, NT-MDT, Russia). A 632.8 nm He–Ne laser (Spectra-Physics, Germany) served as the excitation source. Both excitation and signal collection were achieved through a 100× objective lens with a numerical aperture (NA) of 0.7 (Mitutoyo, Japan). Stokes TERS spectra were recorded using a spectrometer equipped with a 600 lines/mm diffraction grating. It should be noted that, as reported in a previous study, the thermal drift of our setup under laser illumination was measured to be 0.02–0.03 nm s^–1^.^76^ All hyperspectral TERS measurements were performed under the same top-illumination/detection geometry, which minimizes optical geometry-dependent differences between data sets. Therefore, while mode-dependent angular emission or collection effects may affect absolute intensity,^77,78^ they are not expected to alter the observed relative spectral evolution in our experimental configuration.

### XPS Measurements

X-ray photoelectron spectroscopy (XPS) was conducted using a Sigma 2 instrument from Thermo Fisher Scientific. The instrument included an ultrahigh vacuum (UHV) chamber maintained below 5 × 10^–8^ mbar, a nonmonochromatic 200 W Al K_α_ source, an Alpha 110 hemispherical analyzer, and a seven-channel electron multiplier. Na_2_SO_3_ and Na_2_SO_4_ powders were loaded into Al sample holders, while the Au(111) films with 4-MPBA samples before and after UV-ozone treatment were directly mounted on the sample stage with a Cu clamp. High-resolution Au 4f, S 2p, and C 1s spectra were collected with a pass energy of 20 eV, a step size of 0.1 eV, and a dwell time of 50 ms. For the B 1s high-resolution spectra, the acquisition parameters were identical, except that a pass energy of 35 eV was used. All XPS data were analyzed using the CasaXPS software.^72^ Charge corrections were applied by aligning the C 1s peak of adventitious carbon to 284.8 eV for the Na_2_SO_3_ and Na_2_SO_4_ powder references, while the 4-MPBA SAMs on Au(111) samples before and after UV-ozone treatment were charge-corrected versus Au 4f_7/2_ at 84.0 eV. Fitting of the S 2p signal was performed using a Shirley background. The spin–orbit coupling of the S 2p doublet was constrained to 1.16 eV, and the area of the 2p_1/2_ component was fixed to 0.511 times that of the corresponding 2p_3/2_ component. The LA­(1.45, 1.39, 117) line shape of the S 2p components was determined on the Na_2_SO_4_ reference utilizing the CasaXPS optimization procedure and assumed to be valid also for all S 2p components of the other samples. For B 1s, a linear background was applied. The C 1s intensity was normalized to the height of the Au 4f_7/2_ peak with respect to the background intensity at lower binding energy.

### Temperature-Programmed Desorption–Mass Spectrometry (TPD-MS) Measurements

Active capillary plasma ionization was employed due to its high sensitivity toward both polar and nonpolar compounds, enabling the detection of species desorbed from sample surfaces at atmospheric pressure. The plasma was generated using a dielectric barrier discharge configuration, which incorporated two sets of four electrodes isolated by a glass capillary. An AC high voltage (2.6 kVpp, 40 kHz) was supplied to the outer electrode, with the inner electrode maintained at ground potential. The ionization source was directly coupled to an LTQ Orbitrap mass spectrometer (Thermo Fisher Scientific), ensuring nearly 100% ion transmission efficiency. Mass spectral data were collected in positive and negative ion modes across a range of *m*/*z* 50–800, at a resolving power of 30,000 (at *m*/*z* 400). The desorption chamber used for TPD-MS measurement was made of glass, with heating elements placed beneath the sample. The temperature was linearly increased between 300 and 500 K at a rate of around 20 K min^–1^. This chamber was directly connected to the ionization source.

### ESI–Mass Spectrometry Measurement

Mass spectrometry measurements were performed on a Synapt G2 (Waters) instrument equipped with a nanoelectrospray ionization (nESI) source. Samples were prepared in 200 mM ammonium acetate buffer (pH ∼ 7.4). The solution corresponding to the blue trace in Figure 3d corresponds to an untreated 4-MPBA reference solution at 10–20 μM, whereas the red trace corresponds to the ammonium acetate extract obtained by rinsing the UV-ozone-treated 4-MPBA/Au(111) surface. Samples were introduced using in-house-fabricated borosilicate glass capillaries loaded with approximately 3–5 μL of sample solution and fitted with a platinum wire. Measurements were carried out in negative ionization using V-mode with the *m*/*z* range set to 50–1000. The capillary voltage was set to −1.0 kV. The source temperature was maintained at 28 °C, and the sampling cone was operated at 20 V. The backing pressure was ∼3.5 mbar, with the trap and transfer collision energy set to 5 V. The instrument was operated in resolution mode and externally calibrated with cesium iodide (10 mg/mL).

### DFT Calculations

The thiol proton was removed from the molecular structure of 4-MPBA, and the resulting 4-MPBA thiolate sulfur atom coordinated to a three-Au-atom cluster to approximate the local Au(111) binding environment, was optimized using Gaussian 16 at the PBE0/def2-TZVPP level of theory. The def2-TZVPP basis set was applied to all atoms except gold, for which the SDD pseudopotential was used. The approximated local Au(111) binding environment using a three-Au-atom cluster coordinated to 4-MPBA was employed to maintain computational tractability. Similar cluster-based models have been used in previous TERS studies to reproduce and interpret local molecule–surface vibrational features, including our earlier work.^51^ Although this simplified cluster model does not reproduce the full periodic Au(111) surface, it captures the essential local Au–S anchoring motif and molecule–surface interactions relevant to the observed spectral evolution, while providing a practical balance between computational tractability and mechanistic interpretability.

## Supplementary Material



## Data Availability

The original data used in this publication are made available in a curated data archive at ETH Zurich (https://www.researchcollection.ethz.ch) under the DOI: 10.3929/ethz-c-000795818.

## Notes

STM image and corresponding height profile of Au(111) surface; TERS and far-field Raman spectra of 4-MPBA SAMs on Au(111); not background-subtracted TERS spectra and corresponding background analysis; hyperspectral TERS maps of 4-MPBA under ambient and inert N_2_ conditions; laser-power-dependent TERS spectra of 4-MPBA; hyperspectral TERS maps of 4-MPBA at low and high laser-power irradiation; analysis of the early-stage degradation behavior of 4-MPBA; TERS measurements of reference compounds under high laser power; time-dependent evolution of the S=O stretching band intensity; analysis of the early-stage degradation behavior of 4-MPBA; TERS measurements of reference compounds under high laser power; XPS spectra with binding energy assignments of S 2p (referenced to Na_2_SO_3_ and Na_2_SO_4_) and of B 1s and C 1s for 4-MPBA SAMs on Au(111) before and after UV-ozone treatment; binding energy assignment of S 2p; and schematic of the TPD-MS setup and corresponding TPD-MS data of 4-MPBA on Au(111) (PDF)
