# Supplementary material for: Plasmonic Nanocavity-Induced Degradation Pathway of Boronic Acid Biosensing Interfaces Revealed by In Situ Tip-Enhanced Raman Spectroscopy
Source: ACS Nano. 2026 Jun 23;20(26):18941–51. doi: 10.1021/acsnano.6c05687 (PMC13374562; doi:10.1021/acsnano.6c05687)
Supplement: Supplementary file 1 [file nn6c05687_si_001.pdf]

Supporting Information

Plasmonic Nanocavity-Induced Degradation Pathway of Boronic Acid Biosensing Interfaces Revealed by *In Situ* Tip-Enhanced Raman Spectroscopy

Chengcheng Xu[1†], Yuanzhi Xia[1†], Julia Specht[1], Xiaokang Guan[2], Lyna Bourehil[1], Kim Greis[1], Robin Dürr[1], Victor Mougel[1], Naresh Kumar[1*], Renato Zenobi[1*]

[1] Department of Chemistry and Applied Biosciences, ETH Zurich, 8093 Zurich, Switzerland

[2] Department of Chemistry and the MOE Key Laboratory of Spectrochemical Analysis & Instrumentation, College of Chemistry and Chemical Engineering, Xiamen University, Xiamen 361005, Fujian, China

† Equal contribution

* Email: zenobi@org.chem.ethz.ch, naresh.kumar@org.chem.ethz.ch

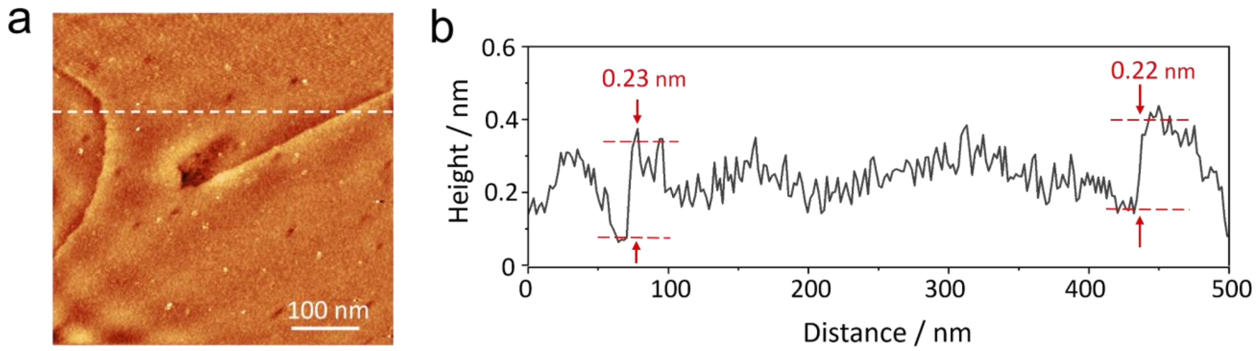

Figure S1. (a) STM topographic image of the clean Au(111) surface used as the substrate for the formation of the 4-MPBA SAM. (b) Height profile along the line marked in panel a, confirming the atomically flat and well-ordered Au(111) terrace structure.

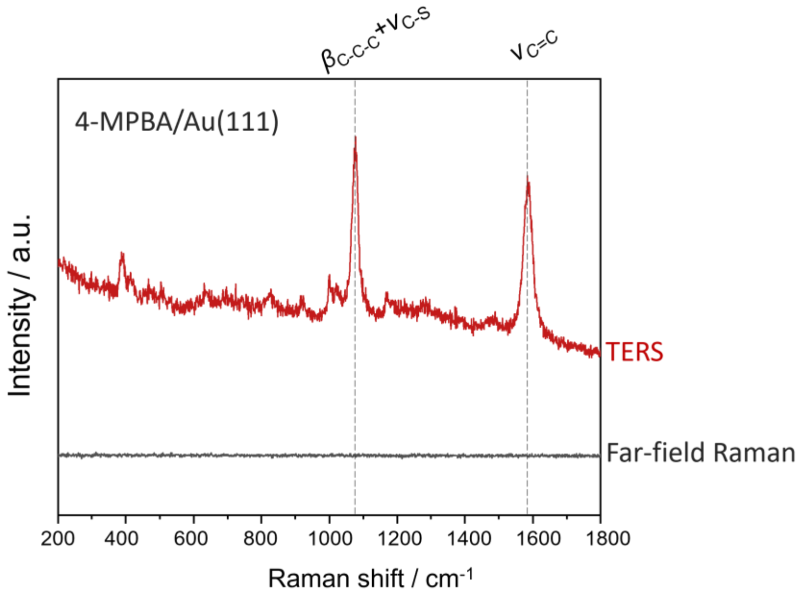

Figure S2. TERS (red) and far-field Raman (grey) spectra of the 4-MPBA SAM on Au(111), recorded with the TERS probe engaged and retracted from the surface, respectively. Laser power: 0.2 mW; integration time: 1 s.

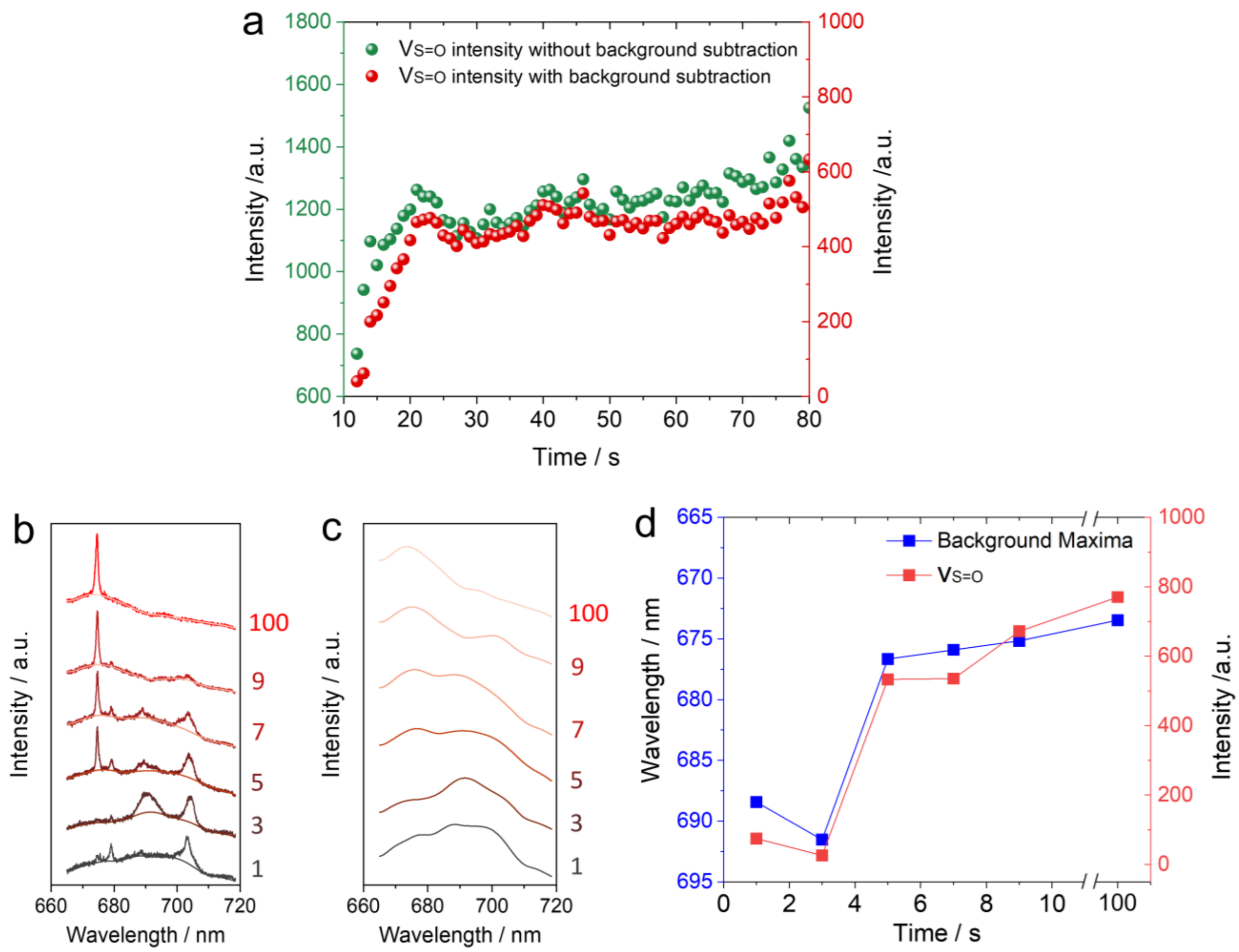

Figure S3. Background analysis of raw, non-background-subtracted TERS spectra acquired during plasmon-induced degradation of 4-MPBA/Au(111). (a) Time-dependent $\nu_{S=O}$ band intensity extracted from raw, non-background-subtracted spectra (green circles, left axis) and from background-subtracted spectra (red circles, right axis). The two traces show the same overall evolution, confirming that the increase in $\nu_{S=O}$ intensity is not introduced by the background-subtraction procedure. (b) Representative raw TERS spectra acquired at selected irradiation times, showing molecular vibrational features superimposed on a broad continuum background. (c) Corresponding broad-background profiles isolated from the spectra shown in panel (b). (d) Comparison of the spectral position of the background maximum (blue squares, left axis) and the $\nu_{S=O}$ band intensity (red squares, right axis) as a function of irradiation time. The background maximum blue-shifts progressively during irradiation and evolves in parallel with the increasing $\nu_{S=O}$ intensity.

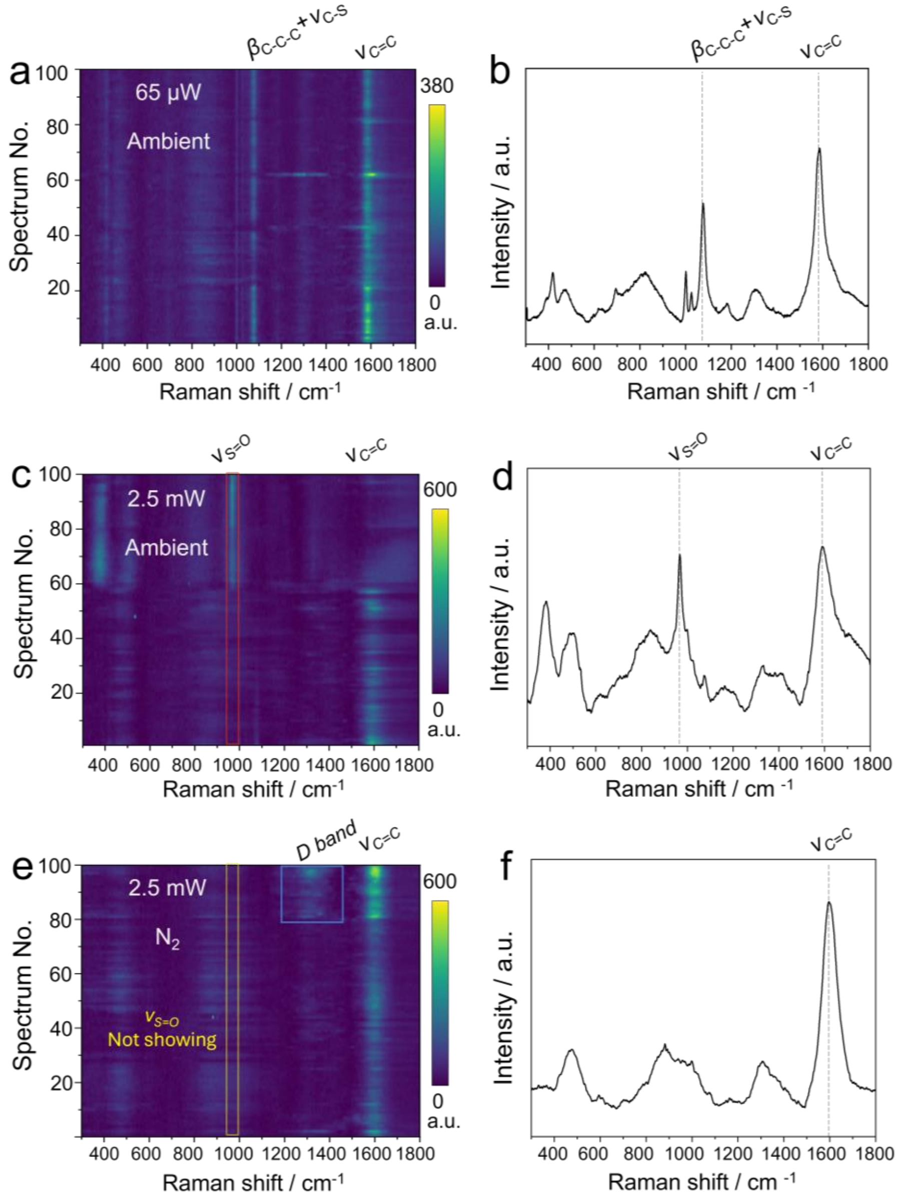

Figure S4. Control TERS measurements of 4-MPBA/Au(111) under ambient and inert N_2 conditions. (a) Waterfall plot of 100 individual TERS spectra acquired under ambient atmosphere at a low laser power of 65 µW with an integration time of 1 s per spectrum. (b) Averaged TERS spectrum derived from the dataset in panel (a), showing the characteristic vibrational features of pristine 4-MPBA. (c) Waterfall plot of 100 individual TERS spectra acquired under ambient atmosphere at 2.5 mW with an integration time of 1 s per spectrum. (d) Averaged TERS spectrum derived from panel (c), showing the emergence of the $\nu_{S=O}$ band associated with oxidized sulfur species. (e) Waterfall plot of 100 individual TERS spectra acquired from a different location on the same sample under inert N_2 conditions at 2.5 mW with an integration time of 1 s per spectrum. (f) Averaged TERS spectrum derived from panel (e). Under N_2, distinct $\nu_{S=O}$ signatures are strongly suppressed, whereas D-/G-band-like features remain observable. These results indicate that oxygen is required for efficient sulfur oxidation, while plasmon-induced intermolecular cross-linking of the 4-MPBA adlayer can still proceed under oxygen-limited conditions. These results are in agreement with previous reports of oxygen-assisted oxidation of thiolate SAMs on Au surfaces.[1,2]

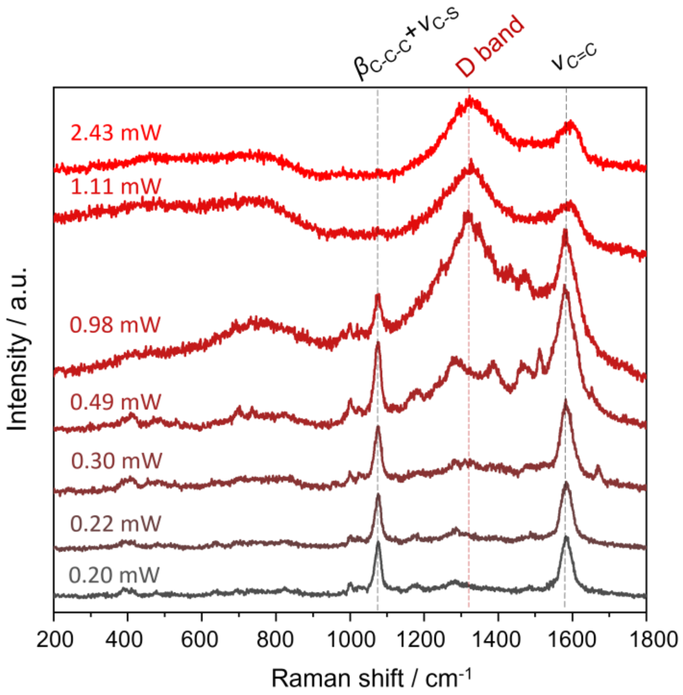

Figure S5. Laser power-dependent TERS spectra of 4-MPBA/Au(111). The laser power was varied from 0.20 mW to 2.43 mW, with each spectrum acquired using an integration time of 1 s. The spectra show that the molecular structure of 4-MPBA SAM remains intact at laser powers below 0.3 mW. In contrast, when the laser power exceeds 1.11 mW, the SAM undergoes a transformation into a highly disordered carbonaceous structure, presumably through extensive intermolecular cross-linking.

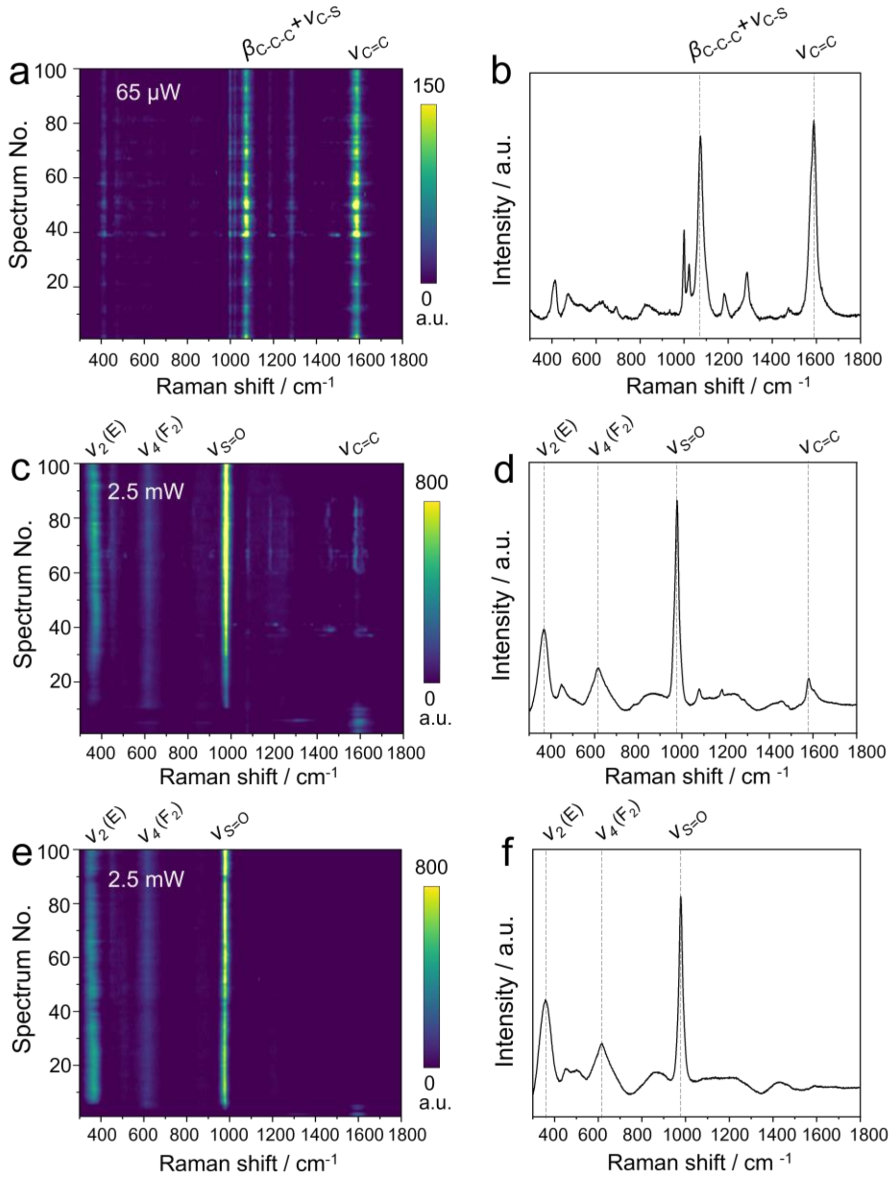

Figure S6. Additional dataset showing reproducibility of the 4-MPBA degradation pathway on Au(111) revealed by TERS. (a) Waterfall plot comprising 100 individual TERS spectra acquired from a hyperspectral TERS map at a laser power of 65 µW. (b) Corresponding averaged TERS spectrum derived from the dataset shown in panel (a). (c) Waterfall plot comprising 100 individual TERS spectra acquired from a hyperspectral TERS map of the same surface region at an increased laser power of 2.5 mW. (d) Corresponding averaged TERS spectrum derived from the data shown in panel (c). (e) Waterfall plot comprising 100 individual TERS spectra acquired from a subsequent hyperspectral TERS map, also recorded at 2.5 mW over the same surface area. (f) Corresponding averaged TERS spectrum derived from the dataset shown in panel (e). The hyperspectral TERS maps in panels (a), (c), and (e) were acquired consecutively under identical conditions. For all maps, the integration time was 1 s per spectrum, the mapped area was 50 × 50 nm², and the step size was 5 nm. TERS measurements performed at a laser power of 2.5 mW reveal a progressive degradation of the 4-MPBA adlayer into oxidized sulfur species. The abundance of these oxidized sulfur species increases systematically with prolonged irradiation at this excitation power.

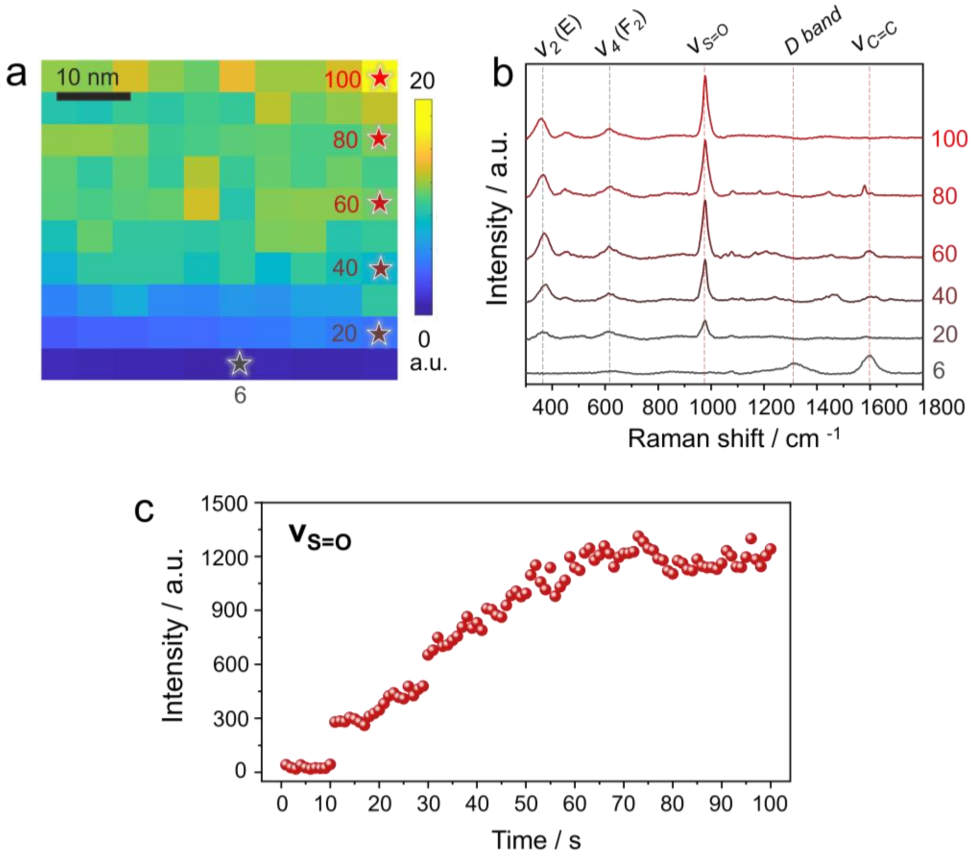

Figure S7. (a) Hyperspectral TERS intensity map of the 978 cm^{-1} vibrational band derived from the dataset shown in Figure S6c. (b) Six representative TERS spectra extracted from distinct pixels within the map in panel (a), revealing the progressive oxidation of 4-MPBA into oxidized sulfur species. (c) Time-dependent evolution of the S=O stretching band intensity extracted from the hyperspectral TERS data in panel (a). This independent dataset reproduces the degradation pathway observed in Figure 2: the 4-MPBA adlayer first undergoes deboronation to form a highly cross-linked intermediate structure (pixel 6 in panel (a)), followed by progressive oxidation of the Au-bound sulfur species (pixels 20–100 in panel (a)).

Supplementary Note 1

Analysis of early-stage degradation behavior of 4-MPBA

To investigate the early-stage degradation behavior of 4-MPBA, we analyzed the sequential spectral evolution in two independent hyperspectral TERS datasets (Figures 2e and S7a) following the approach reported by Shen et al.[3] During plasmon-induced degradation of 4-MPBA/Au(111), the $\beta_{C-C-C} + \nu_{C-S}$ band of pristine 4-MPBA decreases, while the $\nu_{S=O}$ band associated with oxidized sulfur species increases. We therefore defined an apparent pristine-molecule fraction as:

$$R(t) = \frac{A_{pristine}(t)}{A_{pristine}(t) + A_{oxS}(t)}$$

where $A_{pristine}$ is the baseline-corrected integrated area of the $\beta_{C-C-C} + \nu_{C-S}$ band of pristine 4-MPBA, and A_{oxS} is the baseline-corrected integrated area of the $\nu_{S=O}$ band of oxidized sulfur species.

For each hyperspectral TERS map, 100 spectra were acquired sequentially with an integration time of 1 s per spectrum. The spectrum number was therefore converted into acquisition time. Because the largest spectral changes occur at the beginning of irradiation, only the first ten spectra were used for fitting. The decay of $R(t)$ was fitted using a single-exponential function:

$$R(t) = R_{\infty} + A\exp(-kt)$$

where k is the apparent early-stage degradation rate constant. This analysis was applied to the two independent datasets shown in Figures 2e and S7a. The corresponding fits are presented in Figure S8a and S8b, which yield apparent degradation rate constants of 0.9143 and 0.6144 s^{-1}, respectively.

We emphasize that these values represent semi-quantitative apparent early-stage rates rather than absolute kinetic constants. Nevertheless, the analysis provides a useful quantitative measure of the rapid initial loss of pristine 4-MPBA relative to the formation of oxidized sulfur species under high-power plasmonic excitation.

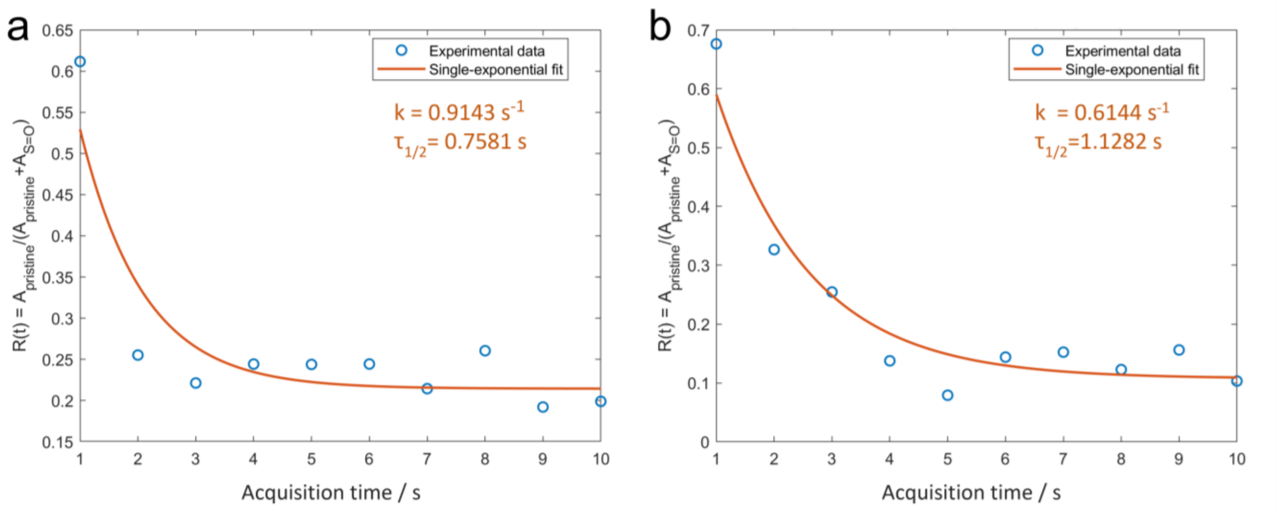

Figure S8. Analysis of the early-stage plasmon-induced degradation of 4-MPBA/Au(111). Single-exponential fits of the apparent pristine-molecule fraction, $R(t) = A_{pristine}/(A_{pristine} + A_{oxS})$, derived from two independent hyperspectral TERS datasets: (a) the dataset shown in Figure 2e and (b) the dataset shown in Figure S7a. $A_{pristine}$ corresponds to the baseline-corrected integrated area of the $\beta_{C–C–C} + \nu_{C–S}$ band of pristine 4-MPBA, while A_{oxS} corresponds to the baseline-corrected integrated area of the $\nu_{S=O}$ band associated with oxidized sulfur species. The first ten sequentially acquired spectra were used for fitting, with the spectrum number converted into acquisition time based on an integration time of 1 s per spectrum. The fitted apparent early-stage degradation rate constants are 0.9143 s^{-1} for the dataset in Figure 2e and 0.6144 s^{-1} for the dataset in Figure S7a.

Supplementary Note 2

TERS measurements of thiophenol and 4-BPT SAMs on Au(111) under high laser power

To test whether plasmon-induced sulfur oxidation is a general response of thiolated SAMs or a molecule-specific degradation channel of 4-MPBA, we performed additional control TERS measurements on thiophenol and 4-BPT SAMs on Au(111) under the same hyperspectral imaging conditions used for the 4-MPBA experiments (Figures S9 and S10 below). These molecules were selected to isolate two structural factors relevant to the 4-MPBA degradation pathway: thiophenol serves as a boron-free phenylthiolate analogue, whereas 4-BPT represents a more extended aromatic thiolate expected to form a more densely packed and π-stabilized monolayer. Under high-power irradiation, thiophenol exhibited broad D-/G-band-like features together with weak S=O stretching signatures, indicating limited sulfur oxidation following plasmon-induced molecular cross-linking. In contrast, 4-BPT retained its characteristic molecular vibrational features and showed no clear S=O band under identical irradiation conditions. These results demonstrate that sulfur oxidation is not unique to 4-MPBA, but it is also not a universal consequence of high-power TERS irradiation of thiolated SAMs. Instead, the efficiency of sulfur oxidation is likely influenced by molecular structure, SAM packing/order, and accessibility of the Au–S interfacial region to oxygen-derived reactive species.[4,5] The full degradation sequence observed for 4-MPBA, deboronation, intermolecular cross-linking, sulfur oxidation, and eventual C–S bond cleavage, therefore appears to be promoted by the boronic acid functionality and the formation of structurally disordered cross-linked intermediates. This molecule-dependent behavior is consistent with previous TERS observations showing oxidized sulfur signatures for some, but not all, thiolated SAMs under comparable irradiation conditions.[6]

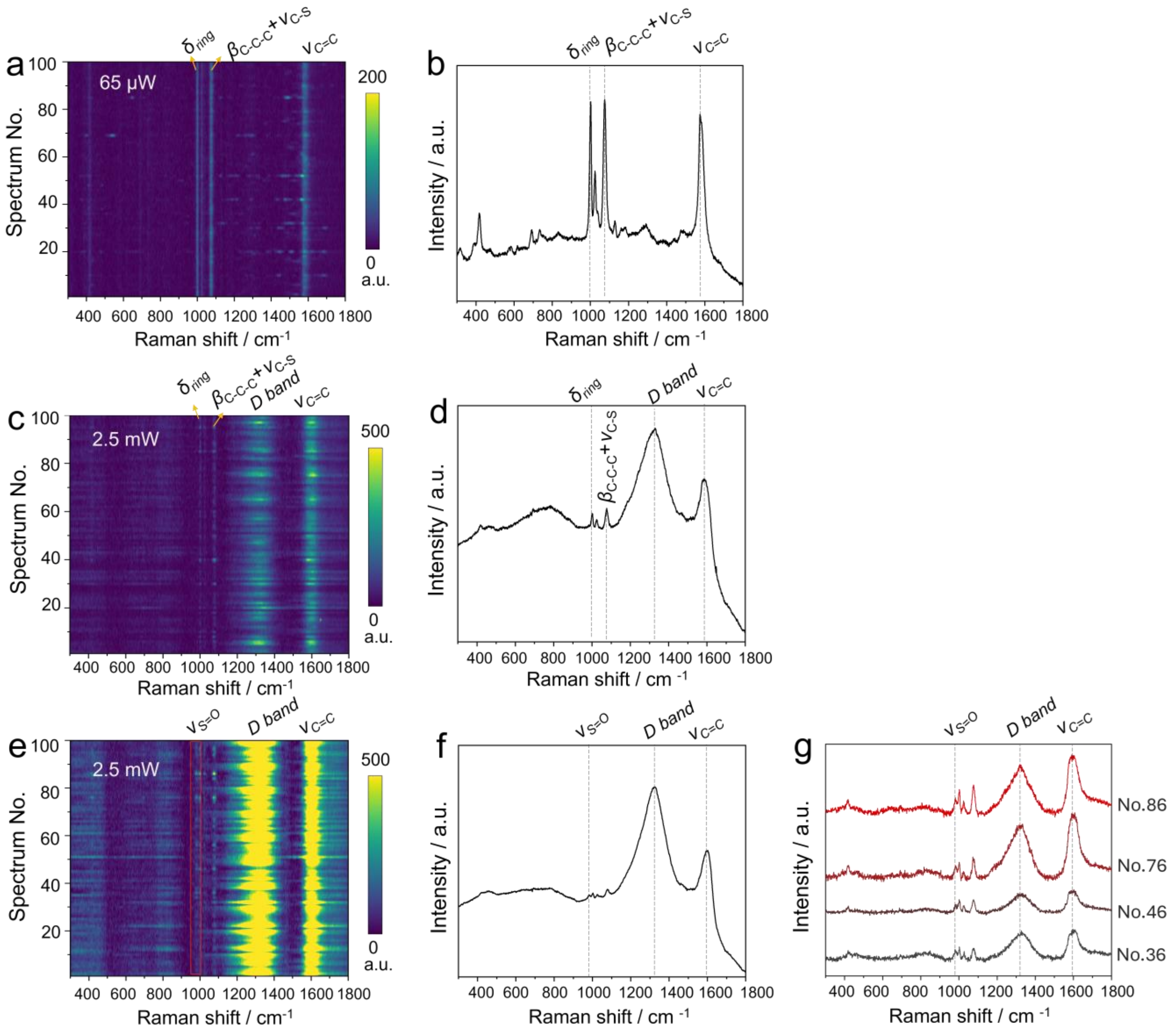

Figure S9. TERS measurements of thiophenol SAMs on Au(111) under the same hyperspectral imaging conditions used for the 4-MPBA degradation experiments. (a) Waterfall plot of 100 individual TERS spectra acquired at a low laser power of 65 μW. (b) Averaged TERS spectrum derived from the dataset in panel (a). (c) Waterfall plot of 100 individual TERS spectra acquired from the same surface region at a high laser power of 2.5 mW. (d) Averaged TERS spectrum derived from the dataset in panel (c). (e) Waterfall plot of a subsequent hyperspectral TERS map acquired over the same surface region at 2.5 mW. (f) Averaged TERS spectrum derived from the dataset in panel (e). (g) Representative spectra selected from panel (e). Upon sustained high-power irradiation, thiophenol exhibits broad D-/G-band-like features, indicating plasmon-induced formation of cross-linked or disordered carbonaceous species, together with weak S=O stretching signatures assigned to oxidized sulfur species. Compared with 4-MPBA, thiophenol therefore undergoes only limited sulfur oxidation under identical TERS irradiation conditions.

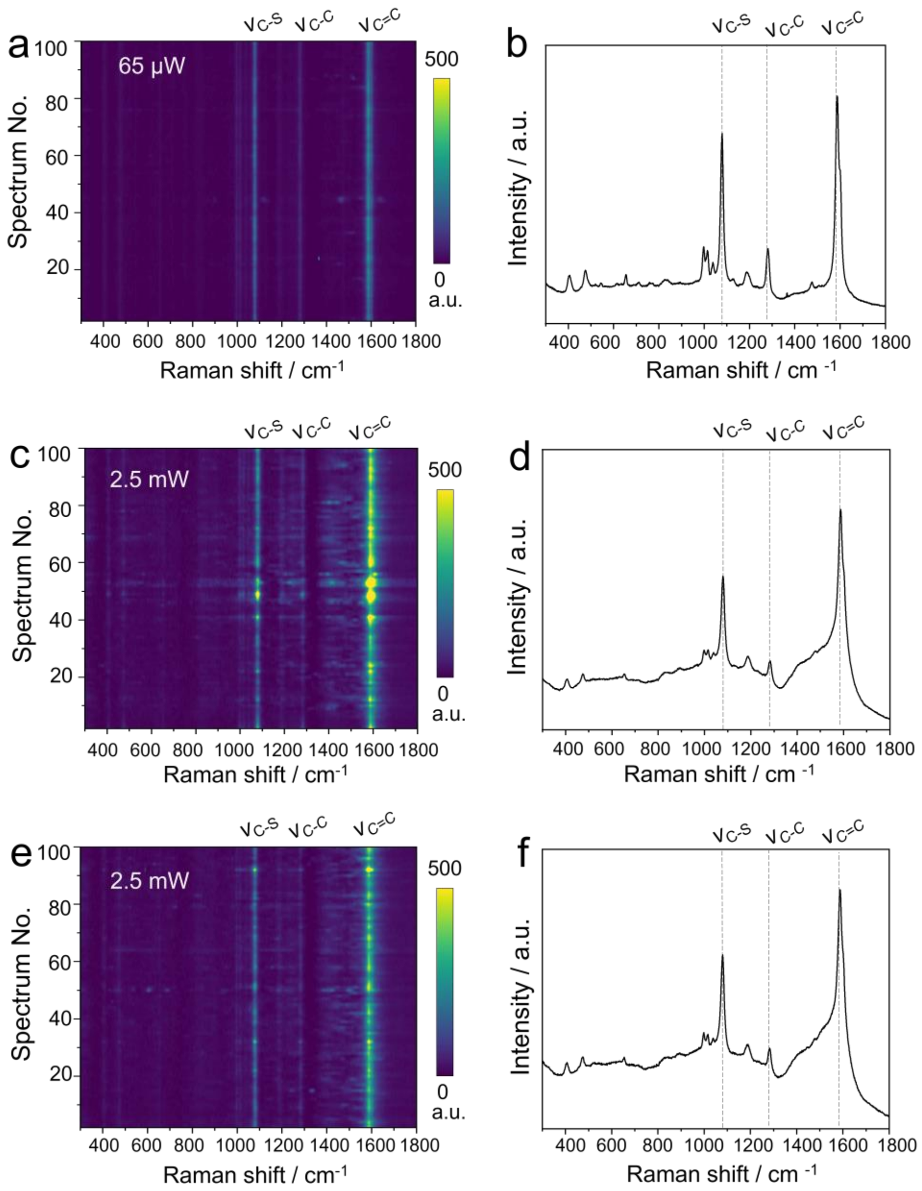

Figure S10. TERS measurements of 4-BPT SAMs on Au(111) under the same hyperspectral imaging conditions used for the 4-MPBA degradation experiments. (a) Waterfall plot of 100 individual TERS spectra acquired at a low laser power of 65 µW. (b) Averaged TERS spectrum derived from the dataset in panel (a). (c) Waterfall plot of 100 individual TERS spectra acquired from the same surface region at a high laser power of 2.5 mW. (d) Averaged TERS spectrum derived from the dataset in panel (c). (e) Waterfall plot of a subsequent hyperspectral TERS map acquired over the same surface region at 2.5 mW. (f) Averaged TERS spectrum derived from the dataset in panel (e). In contrast to 4-MPBA and thiophenol, 4-BPT retains its characteristic molecular vibrational features and shows no clear emergence of an S=O stretching band under high-power irradiation. These results indicate that plasmon-induced sulfur oxidation is strongly suppressed for 4-BPT under the present conditions, highlighting the molecule-specific nature of the degradation behavior of thiolated SAMs.

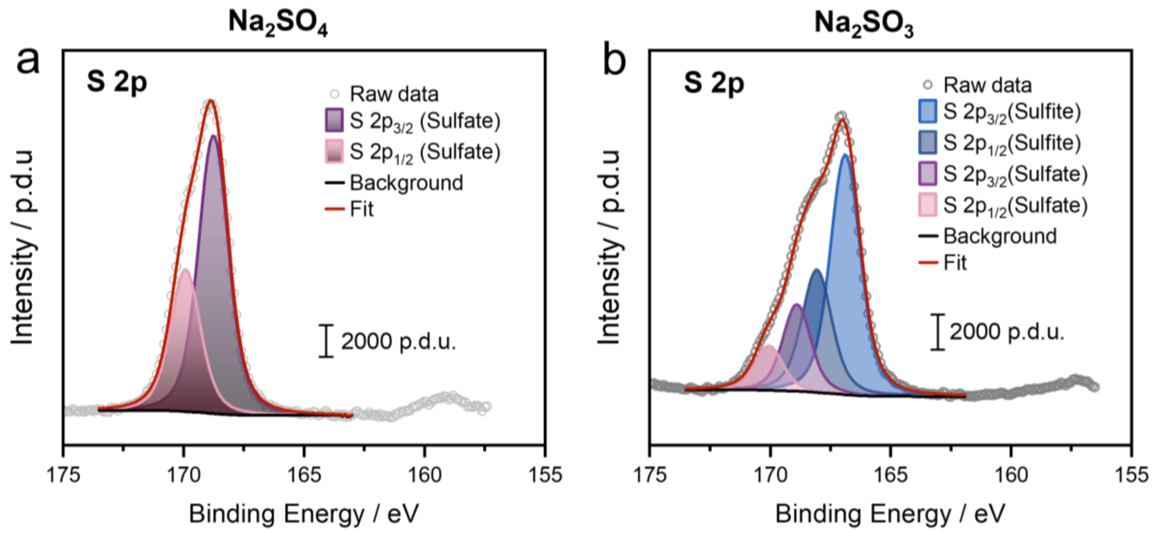

Figure S11. High-resolution S 2p XPS spectra of (a) Na_2SO_4 and (b) Na_2SO_3 reference compounds. For Na_2SO_4, the S 2p doublet corresponding to SO_4^{2-} is observed at binding energies of 168.8 eV (S $2p_{3/2}$) and 169.9 eV (S $2p_{1/2}$), in good agreement with literature values.[7] In contrast, Na_2SO_3 exhibits a distinct S 2p doublet assigned to SO_3^{2-}, with S $2p_{3/2}$ and S $2p_{1/2}$ components located at 166.9 and 168.1 eV, respectively. Notably, the spectrum of Na_2SO_3 also shows a significant contribution at higher binding energies (S $2p_{3/2}$ at 168.9 eV and S $2p_{1/2}$ at 170.1 eV), indicative of partial oxidation to sulfate species. These binding energies closely match those observed for SO_4^{2-} in the Na_2SO_4 reference. Importantly, no changes in the S 2p spectral envelope were observed during XPS acquisition, confirming the absence of X-ray–induced chemical transformations.

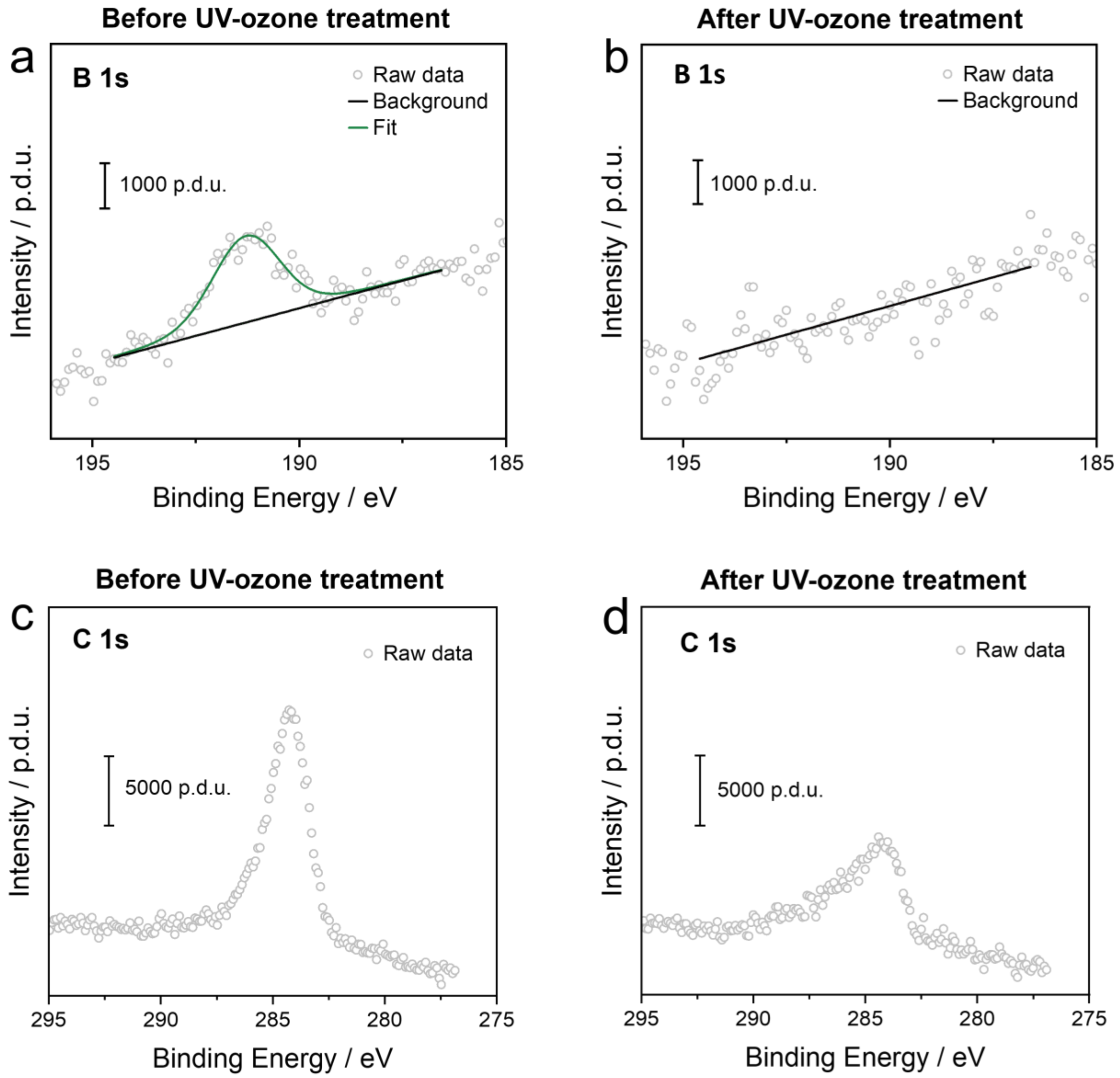

Figure S12. High-resolution B 1s XPS spectra of 4-MPBA SAMs on Au(111) (a) before and (b) after UV–ozone treatment. Prior to UV–ozone exposure, a distinct B 1s signal is observed, indicating an intact 4-MPBA SAM on the Au(111) surface. In contrast, the B 1s signal is absent after UV–ozone treatment, consistent with deboronation during 4-MPBA degradation. High-resolution C 1s XPS spectra of 4-MPBA SAMs on Au(111) before (c) and after (d) UV–ozone treatment. Comparison of the normalized C 1s spectra reveals a substantial decrease in signal intensity following UV–ozone exposure, consistent with C–S bond cleavage and a corresponding reduction in carbon-containing species on the Au(111) surface.

Table S1. Binding energy assignments of surface species identified by XPS for reference samples and 4-MPBA/Au(111) with and without UV-ozone treatment.

Sample	Binding Energy	FWHM	Species	Residual STD
Na_2SO_4	S $2p_{3/2}$: 168.8<br>S $2p_{1/2}$: 169.9	1.46	SO_4^{2-}	1.026
Na_2SO_3	S $2p_{3/2}$: 166.9<br>S $2p_{1/2}$: 168.1	1.46	SO_3^{2-}	1.151
	S $2p_{3/2}$: 168.9<br>S $2p_{1/2}$: 170.1	1.36	SO_4^{2-}	
4-MPBA/Au(111)	S $2p_{3/2}$: 162.0<br>S $2p_{1/2}$: 163.1	1.36	Au-S	0.7726
	S $2p_{3/2}$: 163.6<br>S $2p_{1/2}$: 164.8	1.30	Disulfide, R-SH	
UV-ozone treated 4-MPBA/Au(111)	S $2p_{3/2}$: 168.3<br>S $2p_{1/2}$: 169.5	1.70	SO_4^{x-}	0.7317

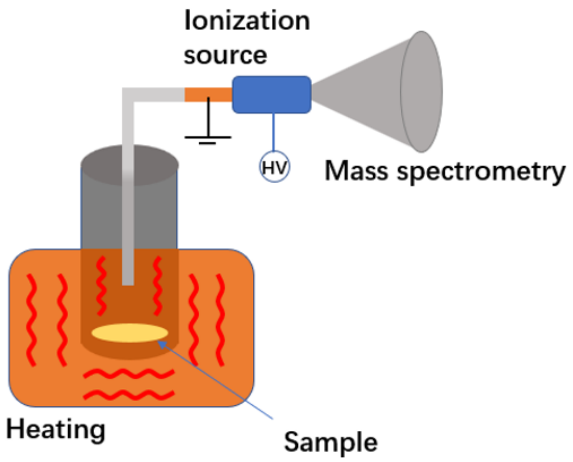

Figure S13. Schematic of the TPD–MS setup. The sample is heated in a glass desorption chamber according to a programmed temperature ramp, and volatilized/desorbed species are transported to the MS inlet for analysis. An AC high voltage (2.6 kVpp, 40 kHz) applied to the outer electrode with the inner electrode grounded generates a dielectric-barrier discharge for ionization of the desorbed species.

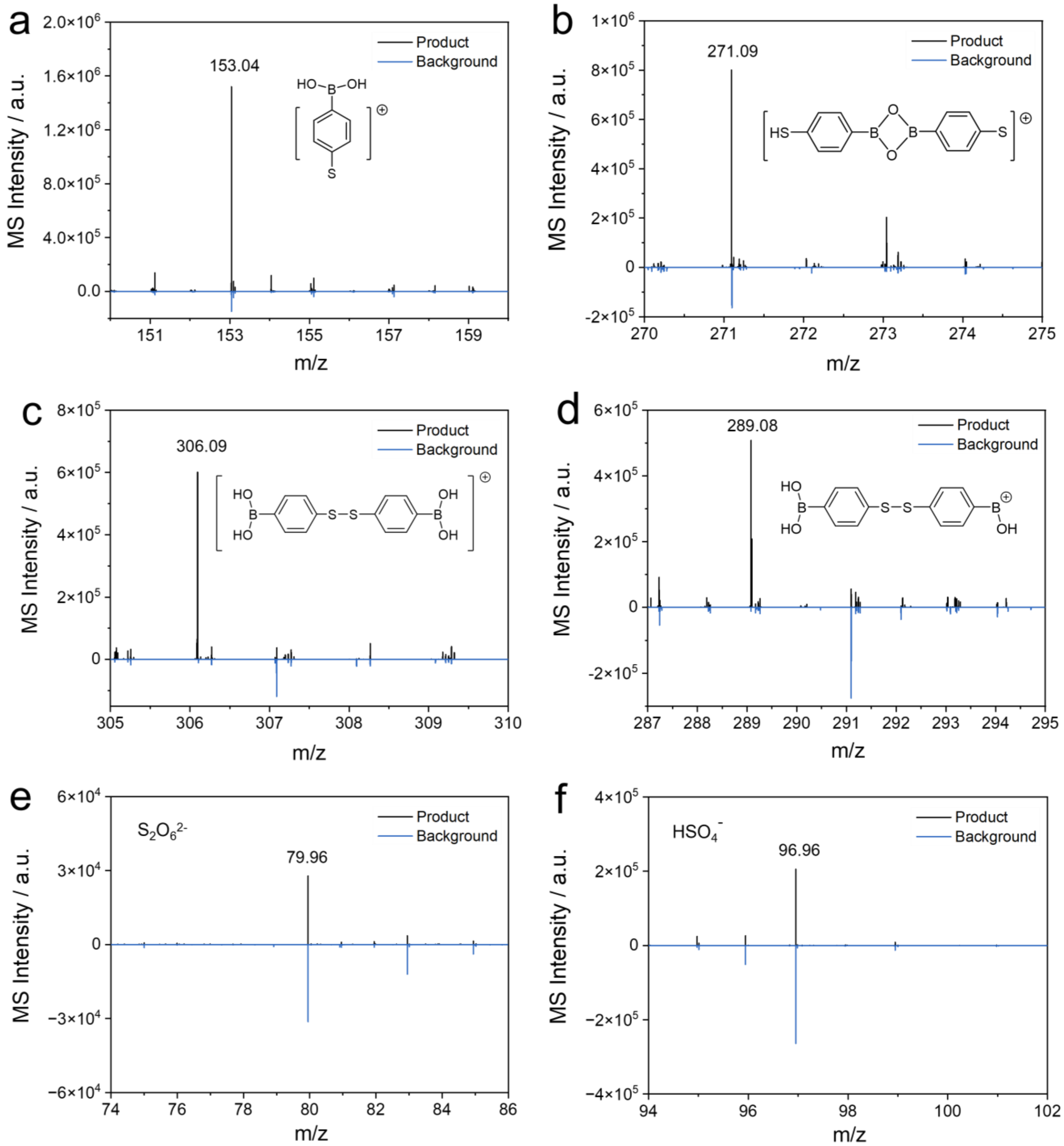

Figure S14. TPD–MS spectra recorded at 420 K in positive ion mode (a–d) and negative ion mode (e, f), showing the ions desorbed from 4-MPBA/Au(111). Background spectra were acquired from a clean Au(111) surface in the absence of the 4-MPBA SAM. For the $S_2O_6^{2-}$ and HSO_4^- ions, similar signal intensities were observed for both the sample and the background, indicating that these species originate from the ambient environment rather than from the sample.

Supplementary References

(1) Sotthewes, K.; Kap, Ö.; Wu, H.; Thompson, D.; Huskens, J.; Zandvliet, H. J. W. Ordering of Air-Oxidized Decanethiols on Au(111). *J. Phys. Chem. C* **2018**, *122* (15), 8430–8436. https://doi.org/10.1021/acs.jpcc.8b01389.

(2) Willey, T. M.; Vance, A. L.; Van Buuren, T.; Bostedt, C.; Terminello, L. J.; Fadley, C. S. Rapid Degradation of Alkanethiol-Based Self-Assembled Monolayers on Gold in Ambient Laboratory Conditions. *Surf. Sci.* **2005**, *576* (1–3), 188–196. https://doi.org/10.1016/j.susc.2004.12.022.

(3) Shen, Y.; Xu, F.; Wei, L.; Hu, F.; Min, W. Live-Cell Quantitative Imaging of Proteome Degradation by Stimulated Raman Scattering. *Angew. Chem. Int. Ed.* **2014**, *53* (22), 5596–5599. https://doi.org/10.1002/anie.201310725.

(4) Hutt, D. A.; Leggett, G. J. Influence of Adsorbate Ordering on Rates of UV Photooxidation of Self-Assembled Monolayers. *J. Phys. Chem.* **1996**, *100* (16), 6657–6662. https://doi.org/10.1021/jp952734h.

(5) Schoenfisch, M. H.; Pemberton, J. E. Air Stability of Alkanethiol Self-Assembled Monolayers on Silver and Gold Surfaces. *J. Am. Chem. Soc.* **1998**, *120* (18), 4502–4513. https://doi.org/10.1021/ja974301t.

(6) Szczerbiński, J.; Gyr, L.; Kaeslin, J.; Zenobi, R. Plasmon-Driven Photocatalysis Leads to Products Known from E-Beam and X-Ray-Induced Surface Chemistry. *Nano Lett.* **2018**, *18* (11), 6740–6749. https://doi.org/10.1021/acs.nanolett.8b02426.

(7) Wahlqvist, M.; Shchukarev, A. XPS Spectra and Electronic Structure of Group IA Sulfates. *J. Electron Spectrosc. Relat. Phenom.* **2007**, *156–158*, 310–314. https://doi.org/10.1016/j.elspec.2006.11.032.
